# Anti-Inflammatory Effects of Nutritionally Relevant Concentrations of Oleuropein and Hydroxytyrosol on Peripheral Blood Mononuclear Cells: An Age-Related Analysis

**DOI:** 10.3390/ijms241311029

**Published:** 2023-07-03

**Authors:** Fanny Pojero, Francesco Gervasi, Salvatore Davide Fiore, Anna Aiello, Sonia Bonacci, Rosalia Caldarella, Alessandro Attanzio, Giuseppina Candore, Calogero Caruso, Mattia Emanuela Ligotti, Antonio Procopio, Ignazio Restivo, Luisa Tesoriere, Mario Allegra, Giulia Accardi

**Affiliations:** 1 Laboratory of Immunopathology and Immunosenescence, Department of Biomedicine, Neurosciences and Advanced Technologies, University of Palermo, 90133 Palermo, Italy; fanny.pojero@gmail.com (F.P.); anna.aiello@unipa.it (A.A.); giuseppina.candore@unipa.it (G.C.); calogero.caruso@unipa.it (C.C.); mattiaemanuela.ligotti@unipa.it (M.E.L.); giulia.accardi@unipa.it (G.A.); 2 Department of Biological, Chemical and Pharmaceutical Sciences and Technologies, University of Palermo, 90123 Palermo, Italy; fioredavide0@gmail.com (S.D.F.); alessandro.attanzio@unipa.it (A.A.); ignazio.restivo@unipa.it (I.R.); luisa.tesoriere@unipa.it (L.T.); 3 Specialistic Oncology Laboratory Unit, ARNAS Hospitals Civico Di Cristina e Benfratelli, 90127 Palermo, Italy; francesco.gervasi1@gmail.com; 4 Department of Health Sciences, University Magna Graecia of Catanzaro, 88100 Catanzaro, Italy; s.bonacci@unicz.it (S.B.); procopio@unicz.it (A.P.); 5 Department of Laboratory Medicine, “P. Giaccone” University Hospital, 90127 Palermo, Italy; rosalia.caldarella@policlinico.pa.it

**Keywords:** oleuropein, hydroxytyrosol, PBMC, LPS, inflammaging

## Abstract

Immunosenescence and inflammaging facilitate the insurgence of chronic diseases. The Mediterranean diet is a non-invasive intervention to improve the chronic low-grade inflammatory status associated with aging. Olive oil oleuropein (OLE) and hydroxytyrosol (HT) demonstrated a controversial modulatory action on inflammation in vitro when tested at concentrations exceeding those detectable in human plasma. We studied the potential anti-inflammatory effects of OLE and HT at nutritionally relevant concentrations on peripheral blood mononuclear cells (PBMCs) as regards cell viability, frequency of leukocyte subsets, and cytokine release, performing an age-focused analysis on two groups of subjects: Adult (age 18–64 years) and Senior (age ≥ 65 years). OLE and HT were used alone or as a pre-treatment before challenging PBMCs with lipopolysaccharide (LPS). Both polyphenols had no effect on cell viability irrespective of LPS, but 5 µM HT had an LPS-like effect on monocytes, reducing the intermediate subset in Adult subjects. OLE and HT had no effect on LPS-triggered release of TNF-α, IL-6 and IL-8, but 5 µM HT reduced IL-10 secretion by PBMCs from Adult vs. Senior group. In summary, nutritionally relevant concentrations of OLE and HT elicit no anti-inflammatory effect and influence the frequency of immune cell subsets with age-related different outcomes.

## 1. Introduction

Control of inflammation becomes challenging as aging progresses, making inflammaging a recognized risk factor for age-associated chronic–degenerative and infectious diseases [1,2,3,4,5,6,7]. In fact, during aging, the concomitant impact of immunosenescence and inflammaging contributes to modelling the conditions favoring the onset of cardiovascular diseases, metabolic diseases, musculoskeletal disorders, neurodegenerative diseases, and cancer, while reducing the ability to face infections and respond to vaccination [1,2,3,4,5,6,7,8,9,10,11,12]. Immunosenescence impacts body homeostasis in terms of the clearance of senescent cells and production of inflammatory cytokines by functional defective/exhausted and senescent immune cells, establishing a strong connection with inflammaging [13,14,15,16,17,18,19,20,21,22,23,24]. Inflammaging is the term used to indicate an aging-related low-grade systemic chronic inflammation, implying the rise of multiple serum inflammatory mediators, as for example, interleukin-1β (IL-1β), IL-6, IL-8, IL-18, and tumor necrosis factor-α (TNF-α), pushed by multiple functional declines involving the impairment of the intestinal barrier, age-associated gut dysbiosis, accumulation of senescence associated secretory phenotype (SASP)-expressing senescent cells, altered reactive oxygen species (ROS) homeostasis linked to mitochondrial impairment, and accumulation of free radicals [7,8,9,11,14,15,16,17,18,25,26,27,28,29,30,31,32,33,34].

The association of chronic disease with age marks the role of lifestyle to ensure successful aging, especially in terms of diet, nutrients intake, and natural (non-nutrient) compounds, in order to prevent age associated pathological conditions. The Mediterranean diet has been proposed as an efficacious and non-invasive intervention to improve the chronic low-grade inflammatory status associated with advanced ages. One of the main pillars of the Mediterranean diet is olive oil, which is rich in phenolic compounds with a demonstrated anti-inflammatory activity [14,35,36,37,38,39,40,41,42,43,44,45,46]. Among all the characterized active molecules, antioxidants oleuropein (OLE) and hydroxytyrosol (HT) demonstrated a controversial modulatory action on inflammation in vitro, with also documented pro-inflammatory effects [37,45,47,48,49,50,51,52]. A key aspect determining OLE and HT pharmacological action is the bioavailability and bioaccessibility of these polyphenols [53,54], with the majority of studies on immune cells [52] analyzing the effect of concentrations that are largely exceeding the maximum achievable plasma values in humans [55,56,57]. 

In this paper, we studied the potential anti-inflammatory effects of OLE and HT at nutritionally relevant concentration on peripheral blood mononuclear cells (PBMCs) as regards cell viability, the frequency of leukocyte subsets, and cytokine release. We performed an age-focused statistical analysis of the results in order to detect differences in the OLE and HT mechanisms of action according to the age of the studied subjects. 

## 2. Results

After selecting PBMCs donors according to the criteria listed in the Section 4, we performed a pooled analysis (collecting data for the whole pool of subjects and comparing results for each assayed condition) and an age-focused analysis, diving our pool of donors into two group: Adult (including subjects aged 18–64 years) and Senior (including subjects whose age was ≥65 years). 

### 2.1. Characteristics of Adult and Senior Groups

Group Adult included 23 subjects (13 females and 10 males), with a median age of 25 years (minimum age 19–maximum age 59); group Senior included 15 subjects (7 females and 8 males), whose median age was 67 years (65–83) (Table 1). As seen in Table 1, the two groups exhibited no statistically significant difference in terms of leukocyte count, while showing a marked difference in serum IL-6 (with higher values recorded for the Senior group) as expected. 

### 2.2. Effects of OLE and HT on Cell Viability

**Figure 1:**
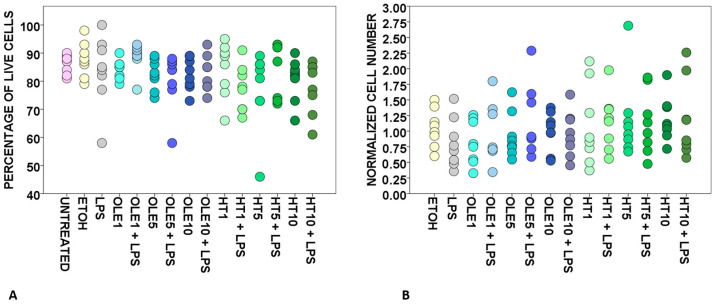
Effects of OLE and HT on PBMC viability (**A**) and normalized PBMC number (**B**). Each dot corresponds to a donor (total: eight donors for each condition, four Adult and four Senior). UNTREATED, cells that received no treatment; ETOH, cells treated only with vehicle (ethanol); LPS, cells treated with 1 μg/mL lipopolysaccharide; OLE1, cells treated with 1 μM oleuropein; OLE1 + LPS, cells pre-treated with 1 μM oleuropein, then incubated with 1 μg/mL LPS; OLE5, cells treated with 5 μM oleuropein; OLE5 + LPS, cells pre-treated with 5 μM oleuropein, then incubated with 1 μg/mL LPS; OLE10, cells treated with 10 μM oleuropein; OLE10 + LPS, cells pre-treated with 10 μM oleuropein, then incubated with 1 μg/mL LPS; HT1, cells treated with 1 μM hydroxytyrosol; HT1 + LPS, cells pre-treated with 1 μM hydroxytyrosol, then incubated with 1 μg/mL LPS; HT5, cells treated with 5 μM hydroxytyrosol; HT5 + LPS, cells pre-treated with 5 μM hydroxytyrosol, then incubated with 1 μg/mL LPS; HT10, cells treated with 10 μM hydroxytyrosol; HT10 + LPS, cells pre-treated with 10 μM hydroxytyrosol, then incubated with 1 μg/mL LPS.

As shown in Figure 1A,B, both OLE and HT add no effect on cell viability and the on normalized cell count of PBMCs cultured with or without the addition of lipopolysaccharide (LPS), as emerged in pooled analysis. 

Group-specific analysis on data collected for the Adult and Senior groups separately also revealed the lack of statistically significant results (Table 2). Moreover, when we tried to detect any condition-related difference in cell viability and normalized cell number comparing data from the Adult and Senior groups for each culture condition, no statistically significant difference emerged, except for 1 μM HT that seemed to improve the percentage of live PBMCs only in Adult vs. Senior subjects (*p* = 0.041) (Table 2). 

**Figure 2:**
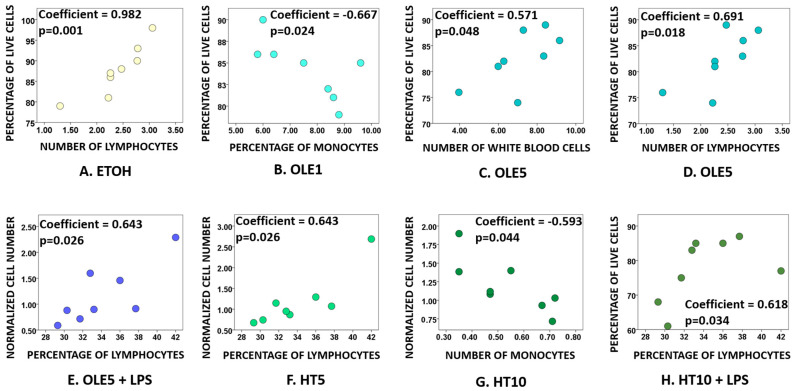
Correlation analysis. Each dot corresponds to a donor (total: eight donors for each condition, four Adult and four Senior). Every diagram depicts a statistically significant correlation detected for PBMCs treated only with ethanol (**A**), PBMCs treated with 1 μM oleuropein (**B**), PBMCs pre-treated with 5 μM oleuropein (**C**,**D**), PBMCs pre-treated with 5 μM oleuropein and then incubated with 1 μg/mL LPS (**E**), PBMCs treated with 5 μM hydroxytyrosol (**F**), PBMCs treated with 10 μM hydroxytyrosol (**G**), and PBMCs pre-treated with 10 μM hydroxytyrosol and then incubated with 1 μg/mL LPS (**H**). ETOH, cells treated only with vehicle (ethanol); OLE1, cells treated with 1 μM oleuropein; OLE5, cells treated with 5 μM oleuropein; OLE5 + LPS, cells pre-treated with 5 μM oleuropein, and then incubated with 1 μg/mL LPS; HT5, cells treated with 5 μM hydroxytyrosol; HT10, cells treated with 10 μM hydroxytyrosol; HT10 + LPS, cells pre-treated with 10 μM hydroxytyrosol, and then incubated with 1 μg/mL LPS.

Correlation analysis performed between the percentage of live PBMCs or the normalized cell count for each condition, age, and value recorded in leukocyte counts (Table 1) revealed the existence of significant correlations between (I) the percentage of live cells treated with the vehicle and the number of lymphocytes (Figure 2A); (II) the percentage of live cells treated 1 μM OLE and the percentage of monocytes (Figure 2B); (III) the percentage of live cells treated with 5 μM OLE and the number of white blood cells and lymphocytes (Figure 2C,D); (IV) the normalized cell number of PBMCs treated with 5 μM OLE together with LPS and the percentage of lymphocytes (Figure 2E); (V) the normalized PBMC number for cells treated with 5 μM HT and the percentage of lymphocytes (Figure 2F); (VI) the normalized cell number of PBMCs treated with 10 μM HT and the number of monocytes (Figure 2G); and (VII) the percentage of live PBMCs treated with 10 μM HT together with LPS and the percentage of lymphocytes (Figure 2H).

Age showed a significant negative correlation with the percentage of live cells in samples treated with LPS only (coefficient = −0.593, *p* = 0.044). Instead, the normalized cell number correlated with age in samples treated with 1 μM OLE (coefficient −0.593, *p* = 0.044), with the normalized cell number declining with age. Intriguingly, culturing cells with 1 μM OLE, the percentage of live PBMCs declines with the concomitant increase in the percentage of monocytes (Figure 2B). However, age and the percentage of monocytes as recorded in leukocyte count had no significant correlation in 1 μM OLE treated samples, whereas age did correlate with the percentage of lymphocytes (coefficient = −0.296, *p* < 0.0005) in untouched PBMCs.

### 2.3. Effects of OLE and HT on the Frequency of Lymphocyte and Monocytes Subsets

**Figure 3:**
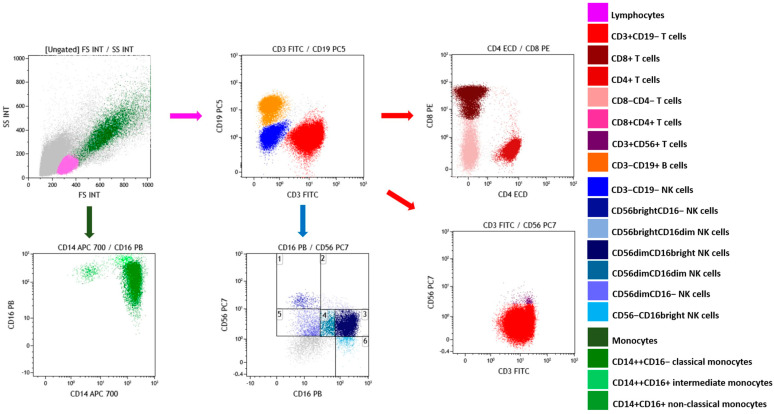
Gating strategy for flow cytometry analysis. Results were collected as percentage of lymphocytes out of total events after exclusion of debris and doublets; CD3+ T cells out of total lymphocytes; CD19+ B cells out of total lymphocytes; CD3−CD19− NK cells out of total lymphocytes; CD4+ T cells out of total CD3+ events; CD8+ T cells out of total CD3+ events; CD4+CD8+ T cells out of total CD3+ events; CD4−CD8− T cells out of total CD3+ events; CD3+CD56+ NKT lymphocytes out of total CD3+ events; CD56brightCD16− NK cells out of total CD3−CD19− events; CD56brightCD16dim NK cells out of total CD3−CD19− events; CD56dimCD16bright NK cells out of total CD3−CD19− events; CD56dimCD16dim NK cells out of total CD3−CD19− events; CD56dimCD16− NK cells out of total CD3−CD19− events; CD56−CD16bright NK cells out of total CD3−CD19− events; monocytes out of total events after exclusion of debris and doublets; CD14++CD16− classical monocytes out of total monocytes; CD14++CD16+ intermediate monocytes out of total monocytes; CD14+CD16+ non-classical monocytes out of total monocytes.

The gating strategy for flow cytometry analysis is depicted in Figure 3 and explained in detail in the caption. 

**Figure 4:**
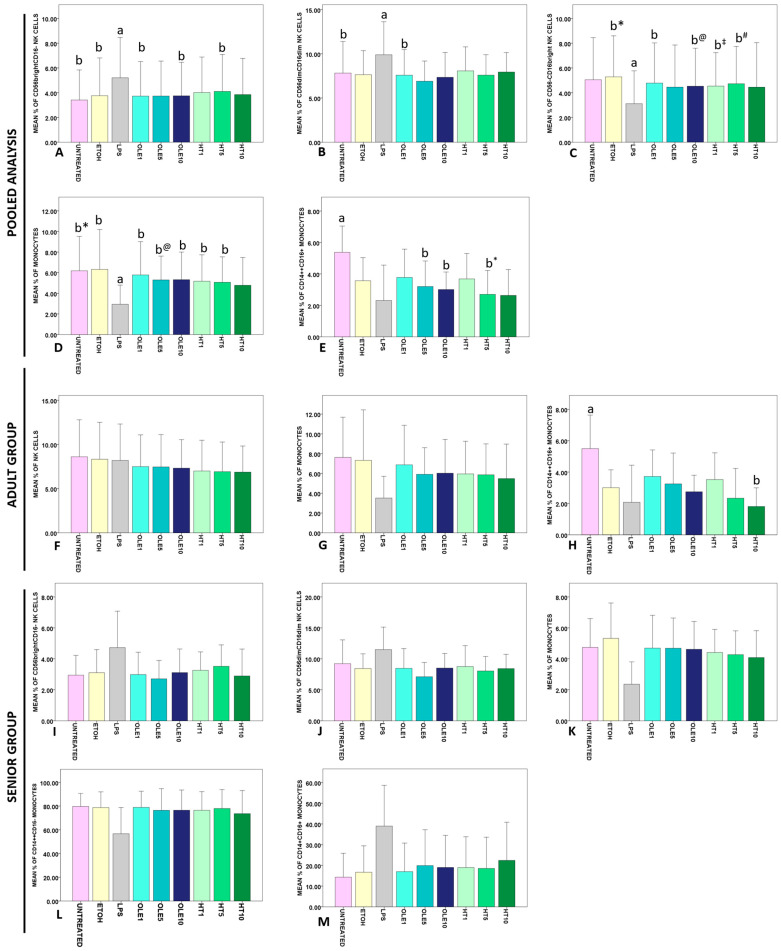
Effects of OLE and HT on the frequency of lymphocyte and monocyte subsets as assayed by flow cytometry on PBMCs. The mean percentages of the studied lymphocyte or monocyte subsets recorded for each experimental condition are shown as bars, whereas error bars represent one standard deviation. Data for pooled analysis (panels (**A**–**E**)) were obtained for 10 donors (5 Adult and 5 Senior). Results are depicted as follows: (**A**) CD56brightCD16− NK cells; (**B**) CD56dimCD16dim NK cells; (**C**) CD56−CD16bright NK cells; (**D**) total monocytes; (**E**) classical CD14++CD16− monocytes. In panel (**C**), * indicates that *p* = 0.003, @ indicates that *p* = 0.006, ‡ indicates that *p* < 0.0005, and # indicates that *p* = 0.007. In panel (**D**), * indicates that *p* = 0.009 and @ indicates that *p* = 0.001. In panel (**E**), * indicates that *p* = 0.003. Data for the Adult group (n = 5) are showed in panels (**F**–**H**): (**F**) NK cells; (**G**) monocytes; (**H**) CD14++CD16+ intermediate monocytes. Data for the Senior group (n = 5) are showed in panels (**I**–**M**): (**I**) CD56brightCD16− NK cells; (**J**) CD56dimCD16dim; (**K**) monocytes; (**L**) CD14++CD16− classical monocytes; (**M**) CD14+CD16+ non-classical monocytes. In each diagram, statistically significant pairwise comparisons (*p* < 0.05) are indicated by different small letters. %, percentage; UNTREATED, cells that received no treatment; ETOH, cells treated only with vehicle (ethanol); LPS, cells treated with 1 μg/mL lipopolysaccharide; OLE1, cells treated with 1 μM oleuropein; OLE5, cells treated with 5 μM oleuropein; OLE10, cells treated with 10 μM oleuropein; HT1, cells treated with 1 μM hydroxytyrosol; HT5, cells treated with 5 μM hydroxytyrosol; HT10, cells treated with 10 μM hydroxytyrosol.

As seen for pooled analysis in Figure 4A–E, incubation with LPS drastically affected the percentage of CD56brightCD16− (*p* < 0.0005), CD56dimCD16dim (*p* < 0.0005) and CD56−CD16bright (*p* = 0.002) NK lymphocytes, and monocytes (*p* < 0.0005). All the assayed treatments caused a contraction of CD14++CD16+ intermediate monocytes (*p* < 0.0005), with statistically significant results detected for OLE5, OLE10, and HT5 vs. UNTREATED PBMCs (Figure 4E). Data that did not reach statistical significance at post hoc comparison and non-significant data are summarized in Figure S1. Comparing the results obtained for Adult subjects with those of Senior subjects for each condition revealed no significant differences. 

As seen in Figure 4F,G, statistical analysis performed on the Adult group demonstrated a reduction of NK (*p* = 0.042) that seems OLE- and HT-dependent, and an LPS-, OLE-, and HT-dependent contraction of the monocyte pool and monocytes (*p* = 0.033), with no significant confirmations at pairwise comparisons. On the contrary, data for CD14++CD16+ intermediate monocytes showed that 5 µM HT significantly reduced the percentage of this subset vs. untreated PBMCs (Figure 4H). Non-significant data for the Adult group are summarized in Figure S2.

Analysis performed on the Senior group suggested an LPS-dependent change in the frequency of CD56brightCD16− (*p* = 0.012) and CD56dimCD16dim (*p* = 0.026) NK cells, monocytes (*p* < 0.0005), and CD14++CD16− classical (*p* = 0.007) and CD14+CD16++ non-classical (*p* = 0.004) monocytes, but no confirmations emerged at pairwise comparisons (Figure 4I–M). Non-significant data for the Senior group are reported in Figure S3. 

### 2.4. Effects of OLE and HT on the Release of Cytokines in the Extracellular Medium

**Figure 5:**
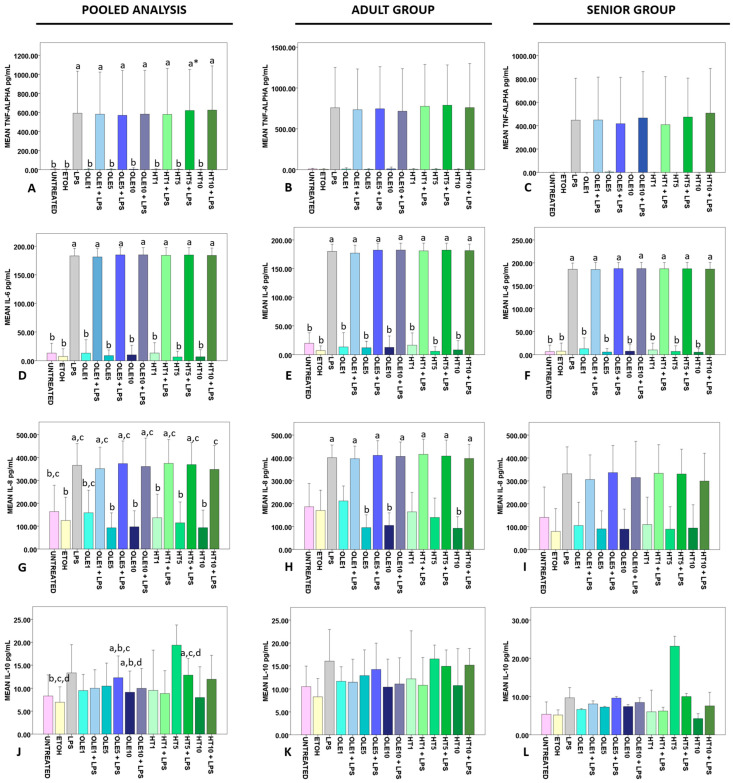
Effects of OLE and HT on cytokine release in the extracellular medium by PBMCs in the presence/absence of LPS. The mean percentages of the studied cytokines recorded for each experimental condition are showed as bars, whereas error bars represent one standard deviation. In each diagram, statistically significant pairwise comparisons (*p* < 0.05) are indicated by different small letters. Data for TNF-α are reported for all the donors (15 subjects, 7 Adult and 8 Senior) in panel (**A**) and in panels (**B**,**C**) for Adult (n = 7) and Senior (n = 8) groups, respectively. In panel (**A**), * indicates that *p* = 0.008 vs. UNTREATED, ETOH, OLE1, OLE5, HT1, HT5, HT10, and *p* = 0.009 vs. OLE10. Results for IL-6 are reported for pooled analysis (18 subjects, 9 Adult and 9 Senior) in diagram (**D**) and in diagrams (**E**,**F**) for Adult (n = 9) and Senior (n = 9) groups, respectively. In panels (**D**–**F**) different small letters correspond to *p* < 0.0005 always. As regards IL-8, data for all the assayed donors (12 subjects, 6 Adult and 6 Senior) are indicated in panel (**G**), whereas results for the Adult (n = 6) and Senior (n = 6) groups are reported in diagrams (**H**,**I**), respectively. For clarity reasons, a detailed summary of statistically significant comparisons is reported in the Table S3 for diagram (**G**) and in Table S4 for diagram (**H**). Data for IL-10 are indicated in panel (**J**) for pooled analysis (7 subjects, 4 Adult and 3 Senior), in panel (**K**) for the Adult group (n = 4), and in panel (**L**) for the Senior group (n = 3). UNTREATED, cells that received no treatment; ETOH, cells treated only with vehicle (ethanol); LPS, cells treated with 1 μg/mL lypopolysaccharide; OLE1, cells treated with 1 μM oleuropein; OLE1 + LPS, cells pre-treated with 1 μM oleuropein, then incubated with 1 μg/mL LPS; OLE5, cells treated with 5 μM oleuropein; OLE5 + LPS, cells pre-treated with 5 μM oleuropein, then incubated with 1 μg/mL LPS; OLE10, cells treated with 10 μM oleuropein; OLE10 + LPS, cells pre-treated with 10 μM oleuropein, then incubated with 1 μg/mL LPS; HT1, cells treated with 1 μM hydroxytyrosol; HT1 + LPS, cells pre-treated with 1 μM hydroxytyrosol, then incubated with 1 μg/mL LPS; HT5, cells treated with 5 μM hydroxytyrosol; HT5 + LPS, cells pre-treated with 5 μM hydroxytyrosol, then incubated with 1 μg/mL LPS; HT10, cells treated with 10 μM hydroxytyrosol; HT10 + LPS, cells pre-treated with 10 μM hydroxytyrosol, then incubated with 1 μg/mL LPS. SD, standard deviation.

Pooled analysis revealed that OLE and HT failed in reducing the amount of LPS-triggered secreted TNF-α in all the tested concentrations (*p* < 0.0005) (Figure 5A). The same statistically significant differences between LPS-challenged and -unchallenged samples were detected when analysis was performed on the Adult (*p* = 0.007) and Senior (*p* = 0.006) groups separately (Figure 5B,C, respectively). Student’s *t*-test revealed that Adult untreated PBMCs were more active in releasing TNF-α than Senior PBMCs (*p* = 0.025). No other statistically significant differences between Adult and Senior subjects emerged.

IL-6 was secreted by LPS-treated PBMCs (irrespectively of OLE and HT treatment) in larger amounts vs. untreated, vehicle only and OLE- or HT-only-treated samples in pooled (*p* < 0.0005) (Figure 5D), Adult (*p* < 0.0005) (Figure 5E), and Senior (*p* < 0.0005) restricted analysis (Figure 5F). No statistically significant correlation was detected with serum IL-6 levels.

Similarly, LPS triggered IL-8 release irrespective of OLE and HT treatment. as detected at pooled analysis (*p* < 0.0005) (Figure 5G), and studying results for the Adult (*p* < 0.0005) (Figure 5H) and Senior (*p* = 0.005) (Figure 5I) groups.

Results for anti-inflammatory IL-10 showed a less-evident LPS-dependent frame at pooled analysis (*p* = 0.011) (Figure 5J), with no statistically significant differences detected when analysis was performed on the Adult group only (Figure 5K). On the contrary, data for the Senior group approached statistical significance (*p* = 0.039), with no significant results at pairwise comparison (Figure 5L), probably as a consequence of the modest sample size. Student’s *t*-test demonstrated that Adult PBMCs were superior to Senior ones in the release of IL-10 after 1 μM OLE (*p* = 0.045) and 10 μM HT + LPS (*p* = 0.044) treatment. Instead, Senior PBMCs were more active than Adult cells in secreting IL-10 as a response to 5 μM HT (*p* = 0.028). No further statistically significant differences emerged in comparing Adult and Senior subjects. 

## 3. Discussion

Among all the factors influencing the pharmacological effect of a given molecule/compound, its bioavailability after administration is crucial. Bioavailability is more than a mere expression of the amount of a given drug that reaches its target; it represents a photograph of the contribution of the administered dose, chosen administration route, rate of adsorption, tissue distribution, and clearance [58].

Evidence about bioavailability of HT and OLE in humans is extremely limited, and a lack of overlap between data obtained from animal models and values reached in humans was reported in the literature about the rate of excretion of both unchanged molecules and metabolites [55,56,57,59,60,61,62,63]. The anti-inflammatory effects of OLE and HT are often assayed in vitro and in vivo using doses that are notably higher than the maximum achievable after ingestion in humans (~4 ng/mL for both OLE and HT) and even higher than the total amount of OLE and HT that would reach systemic circulation in 24 h after extra virgin olive oil assumption or ingestion of supplements [55,56,57,64,65].

In our experimental plan, we decided to use a set of three different concentrations of OLE and HT (1 µM, 5 µM, and 10 µM) that are ten to one hundred-fold lower than commonly used doses to assess OLE and HT anti-inflammatory effect in literature [52]. Blending the experimental assessment of OLE and HT anti-inflammatory activity with building a reliable model of OLE and/or HT (and their metabolites) clearance in vitro is extremely difficult and represents a strong limitation of studies performed on cultured cells/cell lines in literature [52]. However, given that bioavailability of the two polyphenols is estimated as 214 ± 136 ng × h/mL for OLE and 5.3 ng × h/mL for HT [56,64], the concentrations that we tested did not overcome the amount of OLE and HT to which PBMCs would have been exposed in 24 h. 

As regards the chosen inflammatory stimulus and the duration of the exposure, we decided to keep the two most frequent conditions adopted in the literature to assay the anti-inflammatory potential of OLE and HT in vitro, i.e., LPS as an activator of the immune response and 24 h of incubation [52]. Data for HT demonstrated that using HT concomitantly with LPS triggers an inflammatory pattern leading to the increase in the secretion of TNF-α with a poorly understood mechanism that seems also to play a role in the regulation of the release of other inflammatory cytokines [52,66,67]. Since we were interested only in exploring the anti-inflammatory effects of OLE and HT, we decided to use both the polyphenols as a pre-treatment before exposing PBMCs to LPS [52,66,67]. 

To the best of our knowledge, no reports documented the effects of OLE and HT on PBMCs of subjects stratified according to age, thus we hypothesize that age-associated signs of immunosenescence and inflammaging might exert a role in influencing the immune cell answer to OLE and HT. This is an intriguing aspect that deserves further exploration since achieving the rejuvenation of an aged immune system and an improved control of low-grade chronic inflammation is a fundamental goal for a successful aging [1,2,3,9,13,68,69]. 

The effects of OLE and HT on immune cell viability and proliferation are reported as conflicting. HT (but not OLE) may exhibit a cytoprotective effect, being able to preserve DNA from oxidative damage in Jurkatt cells [70] and PBMCs [71]. OLE does not alter cell viability of RAW264.7 and J774A.1 murine cells at concentrations ranging from 5 to 80 μM [72,73] or even higher (100–400 μg/mL) [74]. Similarly, 25–200 μg/mL OLE left human CD4+ cell viability unaltered [75], and human polymorphonuclear cells were unharmed by 320 μg/mL OLE [49]. Moreover, OLE concentrations in the nM and μM ranges improve PBMC viability after γ-irradiation [76]. However, while 50 μM and 100 μM HT protect Jurkat cells from H_2_O_2_ induced apoptosis [77], 20 μM HT reduces the proliferation of THP-1 cells [78], 90 μM HT reduces viability of PBMCs [79], 50–100 μM HT induced apoptosis in HL60 [80] and 10, 75, and 200 μM HT exerted a cytotoxic effect on U937 cells [81]. 

To define the role of nutritionally relevant OLE and HT doses in determining immune cell viability, we tested our set of concentrations for both the polyphenols on PBMCs that were eventually challenged with LPS. We recorded no statistically relevant changes in the percentage of live cells (Figure 1A) or in the normalized cell numbers (Figure 1B), comparing all the culture conditions in pooled analysis. As expected, we also detected no statistically significant differences when comparing matched normalized cell numbers for LPS-unchallenged and -challenged PBMCs (Figure 1B), since only OLE and HT would have accounted for a putative increase in cell counts given that LPS fails in inducing a generalized proliferation of PBMCs in humans [82]. No age-specific effect on cell viability was detected when comparing results for Adult and Senior subjects with Student’s t-test, except for a minimum difference recorded for the percentage of live cells after incubation with 1 μM HT. However, OLE and HT did not offer any specific frame of toxicity or improved cell viability when results were analysed according to subject distribution into the two separate Adult and Senior groups (Table 2), thus leading us to conclude that in comparison with untouched PBMCs or vehicle only- and LPS-treated cells, OLE and HT did not influence cell viability at any of the tested concentrations in both groups. 

In order to detect if other variables may influence the outcome of such an experimental context, we performed a correlation analysis aiming to establish a relationship between cell viability or normalized cell count results and age or leukocyte cell count values reported in Table 1. As expected, vehicle toxicity (even if kept below the safest cut-off levels) [83,84,85,86,87,88,89,90] made the number of lymphocytes a factor influencing the percentage of live cells (Figure 2D). Similarly, the quality of blood samples in terms of percentages of lymphocytes and monocytes emerged as variables correlating with both percentage of live cells and normalized PBMC number (Figure 2). While the effect of isolation methods and freezing/thawing on PBMC viability and survival is well documented [91,92,93,94], it is worth noting that in our pool of subjects, we detected no significant differences in the leukocyte counts, and such a homogeneity might have been reflected in terms of the lack of influence of leukocyte counts on results obtained for PBMC viability and normalized counts. With this prospective in mind, it would be essential to perform a leukocyte count every time the cytotoxicity and/or pro-proliferative effect of OLE and HT should be assayed on PBMCs in order to properly identify statistically significant differences in the sample composition that may affect the final results. 

The evidence supporting a role for OLE and HT in the regulation of immune cell frequency is minimal. Previous studies on mouse spleen lymphocytes demonstrated that 50 μg/mL HT could augment the percentage of CD3+ mouse lymphocytes, and 50, 25, 12.5, and 6.25 μg/mL HT caused an increase in the CD3+CD4−CD8− T cell subset in mouse after 48 h [95]. Moreover, 50, 100, and 200 μg/mL OLE promote the expansion of CD4+CD25+FoxP3+ T regulatory cells (Tregs) in both healthy controls and rheumatoid arthritis patients after 24 h [75]. In order to deepen our knowledge about the effects of nutritionally relevant concentrations of OLE and HT on immune cells in humans, we characterized the main lymphocyte and monocyte subsets by flow cytometry after treatment with both polyphenols and compared the obtained data with fluctuations in the percentage of lymphocyte and monocyte after incubation with LPS. 

We observed that OLE and HT did not induce any significant change in the frequency of T cell subsets and B lymphocytes vs. untreated samples and vehicle only- or LPS-treated PBMCs (Figure S1). In light of the lack of any statistically significant OLE- and HT-induced variations in PBMC numbers mentioned above (Figure 1 and Table 2), the results about flow cytometry assessed distribution of T and B cells were not surprising, even when data for OLE and HT were compared with results collected for LPS challenged PBMCs. In fact, it has already been demonstrated that while promoting proliferation of mouse spleen isolated B cells [96,97], LPS has no effect on human B lymphocyte proliferation in vitro [98]. Moreover, LPS-only-challenged B cells fail in inducing a strong proliferation of mouse CD8+ T cells [99]; LPS priming alters the ability of antigen presenting cells of activating T cells [100]; and LPS suppresses the proliferation of CD4+ and CD8+ T cells in humans [101,102]. 

NK cells deserve a special dissertation. It was previously shown that LPS caused the expansion of NK cells [103] but little is known about the net in vitro effect of LPS on cytotoxic CD56dimCD16bright cells vs. CD56brightCD16dim/− cytokine-secreting NK lymphocyte pool [104,105,106]. The CD56dimCD16dim immature subset [107] and CD56-CD16+ NK cells (whose increase is documented in chronic infections) are even less studied [108,109,110]. At pooled analysis, we noticed no change in the frequency of total NK cells (Figure S1), but LPS caused a significant expansion of CD56brightCD16− (Figure 4A) and CD56dimCD16dim (Figure 4B) NK lymphocytes, while reducing CD56−CD16bright cells (Figure 4C). To the best of our knowledge, this is the first time that an LPS only-dependent expansion of CD56brightCD16− NK cells has been demonstrated, with previous data only referring to proliferation elicited by the combination of LPS and immature dendritic cells [111]. Intriguingly, the effects of LPS on CD56brightCD16− and CD56dimCD16dim seem to depend only on the contribution of the fraction of Senior subjects included in the pooled analysis. In fact, the two mentioned NK subsets were significantly increased after LPS treatment only in the Senior group (Figure 4I,J), while they were unchanged in the Adult group (Figure S2). Whereas the CD56dim NK fraction tends to increase with age [104], the pool of CD56bright NK cells in peripheral blood tends to decrease with aging [104,112]; therefore, such an LPS-dependent increase in CD56bright NK lymphocytes of aged subjects is particularly interesting. Speculating about the possible molecular mechanism explaining the observed LPS effects on Senior group NK cells, we believe that the rise in CD56brightCD16− and CD56dimCD16dim may be difficult to explained by IL-2 action [105,113,114] since exposing PBMCs and monocytes to LPS may suppress IL-2 release [101,115], and IL-2 also decreases during aging [5,116]. Instead, such an expansion might be determined by IL-15 [117,118], which increases with longevity [119] and pushes CD56bright NK proliferation in culture and in human subjects [120,121]. Further investigation in this direction is recommended. In addition, it would be useful to investigate the reasons behind the slight reduction in the percentage of total NK cells that OLE and HT treatments produce only in the Adult group (Figure 4F). A previous paper reports that olive leaf extract administration accounts for an increase in the absolute number of NK cells as measured by flow cytometry [122]. It is possible that when OLE and HT are administrated as a single pharmaceutical agent, they are able to elicit different effects on NK lymphocytes. 

Monocyte subsets exhibit both functional and secretory differences. CD14++CD16− classical monocytes are mainly phagocytic and secrete IL-10 after being challenged with LPS; on the contrary, CD14++CD16+ intermediate and CD14+CD16+ non-classical monocytes are committed to releasing inflammatory cytokines [123,124]. LPS triggers the expansion of TNF-α secreting monocytes [125]; in fact, we observed an increase in cytokine secreting non-classical monocytes [126] that reached statistical significance only in Senior subjects (Figure 4M and Figure S2). However, in pooled analysis, we found that OLE and HT exhibited an effect similar to that of LPS on CD14++CD16+ intermediate monocytes, with a statistically significant reduction in the frequency of this subpopulation vs. UNTREATED PBMCs (Figure 4E). An elegant work by Waller et al. showed that LPS causes the apparent disappearance of intermediate monocytes from the monocyte population, but that this observation is not attributable to an increased cell death rate [127]. This LPS-dependent reduction of intermediate monocytes is related to an LPS-dependent increased shedding of CD14 and CD16, with prolonged LPS incubation causing a superior rate of CD14++CD16− classical monocytes maturation in order to replenish the intermediate monocyte pool [127]. Our results on Senior subjects confirmed the mentioned report in terms of LPS-mediated effects on monocytes, with no changes ascribable to OLE and HT (Figure 4L and Figure S3) [127]. However, in the Adult group, OLE and HT elicited the same effect as LPS, with HT5 eliciting an even more prominent (i.e., statistically significant) reduction of CD14++CD16+ intermediate monocytes. This piece of information is essential since it appears that in adult healthy subjects, OLE and HT are able to mimic the action of LPS at monocyte dynamic level. 

TNF-α, IL-6, and IL-8 are involved in the promotion and maintenance of inflammation at various levels; however, their role in inflammaging is not completely understood. Despite the association of these cytokines with age, chronic diseases, and mortality, their increased levels were sometimes reported as indicators of longevity and successful aging [9,14,116,128]. LPS increases the secretion of TNF-α, IL-6, and IL-8 by human PBMCs [82,129] and monocytes [123,129,130,131,132]; however, data about the effect of OLE and HT on inflammatory cytokine production by human immune cells are extremely scarce, and for IL-8 they are totally absent [52]. Treatment with 10^−4^–10^−7^ M OLE has no effect on TNF-α and IL-6 release in human whole blood culture after LPS stimulation [133], whereas 41 μM HT reduced IL-6 and TNF-α in LPS-challenged human monocytes at both mRNA and protein level [134]. Similarly, 25–100 μM HT produced a reduction in the LPS-dependent secretion of TNF-α in THP1 cells [135]. Evidence arising from murine immune cells are completely different. Treatment with 5–10 μM OLE and HT does not exert any effect of LPS-dependent secretion of TNF-α by mouse RAW264.7 macrophages measured after 18 h of treatment [136], whereas prolonging the experimental conditions up to 24 h [72] and the use of higher doses [74,137] lead to an OLE- and HT-dependent reduction of both TNF-α and IL-6 [maoIL6, ryuIL1]. In another in vitro model, 20–40 μM and 10–40 μM OLE reduce the LPS-triggered secretion of TNF-α and IL-6 by J774A.1 murine macrophages [73]. On the contrary, 12.5 and 6.25 μg/mL HT stimulated TNF-α secretion by mouse spleen lymphocytes [95]. 

In our experimental setting, nutritionally relevant concentrations of OLE and HT did not exert any anti-inflammatory effect on LPS-triggered TNF-α, IL-6, and IL-8 release (Figure 5). Moreover, we did not detect any statistically significant difference in the Adult PBMC ability of releasing IL-6 in the extracellular medium vs. Senior subjects in spite of the confirmed higher cytokine levels in the serum of aged donors (Table 1). This is the first time that IL-8 production has been analyzed on PBMCs after administration of OLE and HT with and without LPS. None of the tested concentrations were able to significantly reduce the amount of IL-8 released in the cell culture medium (Figure 5G), with cells from Adult subjects being generally more active in secreting the cytokine than Senior PBMCs (Figure 5H,I). 

IL-10 is a universally recognized anti-inflammatory mediator, whose role as a longevity factor is uncertain. IL-10 secretion increases with age [128,138,139,140], although aged immune cells are less active in secreting IL-10 after stimulation [141]. In vitro data for anti-inflammatory IL-10 reveal that 50–200 μg/mL OLE induces the production of IL-10 by CD4+ T cells [75], while quantities as low as 1 μM HT amplified the Parietaria antigen-elicited IL-10 secretion by PBMCs [79]. Moreover, 41 μM HT stimulates the synthesis and the release of IL-10 by human monocytes [134]. At pooled analysis, the secretion of IL-10 was increased by treatment with 5 μM HT (Figure 5J), but the observed effect seems to be dependent on the data collected for Senior subjects. In fact, only when Senior PBMCs were treated with 5 μM HT were they able to exceed the production of IL-10 by Adult PBMCs. Given that the same condition accounted for the reduction in the percentage of CD14++CD16+ intermediate monocytes (Figure 4H), and considering that monocytes are a main source of IL-10 [142,143], there may be a correlation between these two observed phenomena. 

Despite the limited number of studied samples, our report has the merit of exploring LPS induced fluctuations in NK and monocyte subsets in PBMC culture, for which little evidence is available. Moreover, our study offers the space to discuss the effects of OLE and HT used at nutritionally relevant concentrations in the light of numerical and functional differences that may be detected in lymphocytes and monocytes during aging. 

In summary, our work demonstrated that OLE and HT elicit no anti-inflammatory effect at nutritionally relevant concentrations. Moreover, OLE and HT exert a different action on the frequency of immune cell subsets that became more evident when analysis is performed according to the age of PBMC donors. It remains to be assessed if this OLE and HT age-related effect on immune cells may have some future applications in terms of promoting the expansion of one or more desired immune population. Previous studies demonstrated that OLE and HT are not stable in plasma, whereas their metabolites may be found in larger quantities in the circulation after OLE and HT ingestion [55,56,63,64] and may account for biological effects on PBMCs that might deserve to be tracked [50,53,144]. In addition, the data reported in this paper encourage the comparison of these results with those triggered by the administration of more stable/bioavailable formulations of OLE and HT [145,146,147,148].

## 4. Materials and Methods

### 4.1. Subjects

A total of 38 subjects (18 males and 20 females) were included in this study. Subjects were dived into two groups: those whose age fell between 18–64 years represented the “Adult” group; those aged ≥ 65 years represented the “Senior” group. All the subjects were free from cancer, hematological malignancies, and acute phase diseases. Subjects who underwent solid organ/bone marrow transplantation or immunomodulating/immunosuppressant therapies in the last six months were excluded from the study. Leukocyte counts were performed at the Department of Laboratory Medicine, “P. Giaccone” University Hospital, Palermo, Italy. The characteristics of both groups are summarized in Table 1. The study was conducted according to the guidelines of the Declaration of Helsinki and approved by the Ethics Committee Palermo 1 (#01/2022, 17 January 2022).

### 4.2. OLE and HT

OLE was extracted from olive leaves of the Coratina cultivar of *Olea europaea* L., as reported previously [149]. In summary, olive leaves were dried for 48 h at 50 °C, milled, and extracted in an Anton Paar Synthos 3000 MW Oven at 800 W (P-controlled mode) for 10 min in water as a solvent. The leaves were filtered, and the solution was dried under pressure. The mixture was treated with acetone and purified from solid residue by filtration. The solution was evaporated under reduced pressure and the crude product was purified by flash chromatography on silica cartridges (CH_2_Cl_2_/MeOH 8:2). OLE was obtained at an HPLC purity of 98%. Analytical data of the pure OLE were compared with data reported in the literature. 

HT was obtained as described in literature [150]. Briefly, glycoside oleuropein was dissolved in 6 M NaOH (5 mL) under argon atmosphere in the dark and stirred for 2 h. The solution was acidified to pH 3 with 2 M HCl and extracted with ethyl acetate. The organic extracts were dried over Na_2_SO_4_ and evaporated in vacuo to afford a residue (30 mg) containing 55% of HT (by HPLC analysis). Purification on silica gel (20:1, *w*/*w*) by elution with CHCl_3_/MeOH (80:20, *v*/*v*) gave a pure standard of HT (5 mg; 0.03 mmol; yield 11%). Spectroscopic data were consistent with those reported in the literature. 

For the experimental procedures on cell cultures, OLE and HT were each resuspended in ethanol and used at the final concentrations of 1 μM, 5 μM and 10 μM. 

### 4.3. Materials

Ficoll-Paque™ PLUS (Cytiva, Marlborough, MA, USA) was used for PBMC isolation.

For culture and assessment of cell viability, RPMI 1640, foetal bovine serum, antibiotics, L-glutamine, sodium pyruvate, MEM non-essential amino acids, and trypan blue were all purchased from Thermo Fisher Scientific (Waltham, MA, USA).

For the assessment of cytokine secretion in cell culture, TNF alpha Human Uncoated ELISA Kit, IL-6 Human Uncoated ELISA Kit, IL-8 Human Uncoated ELISA Kit (all purchased from Thermo Fisher Scientific, Waltham, MA, USA), and ELISA Kit for Interleukin 10 (IL10) by Cloud-Clone Corp. (Katy, TX, USA) were used.

*E. coli* O55:B5 LPS was purchased from Sigma-Aldrich (St. Louis, MO, USA). 

Antibodies used for flow cytometry analysis were all purchased from Beckman Coulter (Brea, CA, USA) and detailed in Table S1.

### 4.4. Blood Samples, PBMC Isolation and Treatment

Venipuncture was performed on each donor after 12 h of fasting in the first hours of the morning. Peripheral blood was collected in EDTA tubes and manipulated within one hour of collection. PBMCs were isolated through Ficoll-Paque™ PLUS stratification according to the manufacturer instructions. Obtained PBMCs were counted in Countess II cell counter (Thermo Fisher Scientific, Waltham, MA, USA) using trypan blue to assess viability and stored at −80 °C or in liquid nitrogen up to their use. 

PBMCs were brought to a final concentration of 5 × 10^5^ cells/mL in RPMI 1640 supplemented with 10% heath-inactivated fetal bovine serum, 1% antibiotics, 1% L-glutamine, 1% sodium pyruvate, and 1% MEM non-essential amino acids and were left untreated (condition “Untreated”), treated with vehicle only (condition “ETOH”, standing for ethanol), or received OLE and HT according to the concentrations listed in the previous paragraph (conditions “OLE1”, “OLE5”, and “OLE10” for 1 μM, 5 μM, and 10 μM OLE, and conditions “HT1”, “HT5”, and “HT10” for 1 μM, 5 μM, and 10 μM HT, respectively). After two hours, PBMCs were left untouched for the following 24 h, or LPS was added to PBMC cultures at the final concentration of 1 μg/mL obtaining the following experimental scenarios: (I) LPS only (condition “LPS”); (II) pre-treatment with 1 μM, 5 μM, and 10 μM OLE followed by LPS addition (conditions “OLE1 + LPS”, “OLE5 + LPS”, and “OLE10 + LPS”, respectively); and (III) pre-treatment with 1 μM, 5 μM, and 10 μM HT followed by incubation with LPS (conditions “HT1 + LPS”, “HT5 + LPS”, and “HT10 + LPS”, respectively). After 24 h, cell culture medium and cultured PBMCs were collected in order to perform the analyses listed in the following paragraphs. 

### 4.5. Assessment of Cell Viability

PBMC viability and numbers were assessed by trypan blue exclusion assay as measured by manual counting and confirmed in Countess II cell counter (Thermo Fisher Scientific, Waltham, MA, USA) in order to avoid the documented limitations of MTT- and MTS- based assays depending on the interferences of phenolic compounds [151,152]. Cell viability was expressed as percentage of live cells out of total counted cells. Cell counts were expressed as normalized values, i.e., the number of live counted cells normalized on the number of cells counted in wells received only medium in order to estimate by how many folds the cell number increases after treatment in comparison with PBMCs left untreated. 

### 4.6. Frequency of Lymphocyte and Monocyte Subsets

Frequency of T, NKT, B, and NK subsets and monocyte subsets (classical, intermediate, and non-classical) in cultured PBMCs was assessed in Navios flow cytometer (Beckman Coulter, Brea, CA, USA) with the settings listed in Table S2, carefully taking into account the documented effects of cryopreservation on PBMCs [153,154,155]. The gating strategy was established as follows: after exclusion of debris and doublets, a first gate was set in the forward scatter (FSC) vs. side scatter (SSC) dot plot on the lymphocyte region; in the CD3 vs. CD19 dot plot, a gate was set on CD3+ T and NKT cells and on CD19+ B cells and on CD3−CD19− NK cells. CD3+ cells were subsequently studied in CD4 vs. CD8 dot plot and in a CD3 vs. CD56 dot plot to determine the frequency of NKT cells. CD3-CD19- NK cells were analyzed in CD16 vs. CD56 dot plot to assess the frequency of NK subpopulations [106,107]. A further gate set in the FSC vs. SSC dot plot helped in the identification of monocytes that were characterized in the CD14 vs. CD16 dot plot [123,124,142]. Data were analyzed with Kaluza C software (https://www.beckman.com/flow-cytometry/software/kaluza-c) (Beckman Coulter, Brea, CA, USA).

### 4.7. Measurement of Cytokine Release in the Extracellular Medium

The concentration of TNF-α, IL-6, IL-8, and IL-10 in the extracellular medium after treatment with OLE and HT with and without LPS was measured with the ELISA kits listed in Section 4.3 according to the manufacturer instructions. 

### 4.8. Statistical Analysis

Data were collected for the whole pool of subjects (and data were explored in the form of “pooled analysis”) and for the two groups—Adult and Senior—separately. Normality of data was assessed graphically and by the Shapiro–Wilk test. Repeated measures ANOVA (with Greenhouse–Geisser correction whenever sphericity could not be assumed) was used for normally distributed data. Student’s *t*-test was used to compare normally distributed data of independent samples. Mann–Whitney U test for two independent samples were used for variables that did not meet the requirements for parametric tests. Kendall tau b correlation coefficient was used to determine the correlation between continuous variables. Statistical significance was set for *p* < 0.05. The analyses were performed through IBM Corp. Released 2013. IBM SPSS Statistics for Windows, Version 22.0. Armonk, NY, USA: IBM Corp.

## Figures and Tables

**Table 1:** Characteristics of the subjects.

	Adult			Senior			*p*
**Gender**							
	**Female**	**Male**	**Total**	**Female**	**Male**	**Total**	
	13	10	23	7	8	15	n.s.
**Age**							
	**Median**	**Minimum**	**Maximum**	**Median**	**Minimum**	**Maximum**	
	25	19	59	67	65	83	<0.0005
**WBC × 10^3^/μL**							
	**Mean**	**S.D.**	**95% C. I.**	**Mean**	**S.D.**	**95% C. I.**	
	7.08	1.56	6.40–7.75	7.27	1.34	6.53–8.01	n.s.
**Lymphocytes × 10^3^/μL**							
	**Mean**	**S.D.**	**95% C. I.**	**Mean**	**S.D.**	**95% C. I.**	
	2.41	0.47	2.20–2.61	2.27	0.37	2.06–2.47	n.s.
**Monocytes × 10^3^/μL**							
	**Mean**	**S.D.**	**95% C. I.**	**Mean**	**S.D.**	**95% C. I.**	
	0.53	0.13	0.47–0.59	0.56	0.15	0.48–0.64	n.s.
**Neutrophils × 10^3^/μL**							
	**Mean**	**S.D.**	**95% C. I.**	**Mean**	**S.D.**	**95% C. I.**	
	3.87	1.30	3.31–4.44	4.22	1.14	3.59–4.86	n.s.
**%Lymphocytes**							
	**Mean**	**S.D.**	**95% C. I.**	**Mean**	**S.D.**	**95% C. I.**	
	34.95	6.38	32.19–37.71	31.84	6.27	28.37–35.31	n.s.
**%Monocytes**							
	**Mean**	**S.D.**	**95% C. I.**	**Mean**	**S.D.**	**95% C. I.**	
	7.58	1.13	7.09–8.07	7.77	1.98	6.67–8.87	n.s.
**%Neutrophils**							
	**Mean**	**S.D.**	**95% C. I.**	**Mean**	**S.D.**	**95% C. I.**	
	54.13	7.54	50.88–57.39	57.25	7.01	53.36–61.13	n.s.
**Serum IL-6**							
	**Median**	**Minimum**	**Maximum**	**Median**	**Minimum**	**Maximum**	
	1.5	1.5	3.41	2.52	1.5	13.6	0.002

WBC, white blood cell count expressed as counted cells × 10^3^/μL; Lymphocytes, number of lymphocytes in peripheral blood samples out of leukocyte count expressed as counted cells × 10^3^/μL; Monocytes, number of monocytes in peripheral blood samples out of leukocyte count expressed as counted cells × 10^3^/μL; Neutrophils, number of neutrophils in peripheral blood samples out of leukocyte count expressed as counted cells × 10^3^/μL; %Lymphocytes, percentage of lymphocytes in peripheral blood samples out of leukocyte count; %Monocytes, percentage of monocytes in peripheral blood samples out of leukocyte count; %Neutrophils, percentage of neutrophils in peripheral blood samples out of leukocyte count; S.D., standard deviation; 95% C. I., 95% confidence interval; n.s., non-significant.

**Table 2:** Effects of OLE and HT on PBMC viability and on normalized number assayed on cells from donors of the Adult and Senior groups.

		Adult (n = 4)	Senior (n = 4)	Student’s *t*-Test
**Percentage of live cells**				
	**Untreated**	86.25 ± 3.59	86 ± 3.65	n.s.
	**ETOH**	87 ± 6.06	88.5 ± 7.05	n.s.
	**LPS**	90 ± 8.12	77.5 ± 14.2	n.s.
	**OLE1**	83.75 ± 4.86	84.75 ± 1.89	n.s.
	**OLE1 + LPS**	86.5 ± 6.56	90.75 ± 1.71	n.s.
	**OLE5**	81.75 ± 4.19	83 ± 6.98	n.s.
	**OLE5 + LPS**	77 ± 13.44	83.75 ± 3.69	n.s.
	**OLE10**	82.5 ± 7.19	82.5 ± 5.07	n.s.
	**OLE10 + LPS**	81.5 ± 6.76	84 ± 6.68	n.s.
	**HT1**	90.75 ± 3.78	77.5 ± 9.47	0.041
	**HT1 + LPS**	78.25 ± 6.24	76.5 ± 10.72	n.s.
	**HT5**	78.25 ± 6.4	76.25 ± 20.27	n.s.
	**HT5 + LPS**	87.5 ± 9.68	81.5 ± 10.15	n.s.
	**HT10**	83.25 ± 7.27	78 ± 8.04	n.s.
	**HT10 + LPS**	78.5 ± 11.71	76.75 ± 7.85	n.s.
Repeated measures ANOVA		n.s.	n.s.	
**Normalized cell number**				
	**ETOH**	1.04 ± 0.27	1.06 ± 0.37	n.s.
	**LPS**	0.85 ± 0.31	0.77 ± 0.51	n.s.
	**OLE1**	0.97 ± 0.24	0.66 ± 0.41	n.s.
	**OLE1 + LPS**	0.92 ± 0.48	0.98 ± 0.54	n.s.
	**OLE5**	0.77 ± 0.16	1.08 ± 0.46	n.s.
	**OLE5 + LPS**	1.21 ± 0.37	1.13 ± 0.79	n.s.
	**OLE10**	1.09 ± 0.39	0.9 ± 0.23	n.s.
	**OLE10 + LPS**	1.06 ± 0.16	0.85 ± 0.51	n.s.
	**HT1**	1.12 ± 0.65	1.04 ± 0.73	n.s.
	**HT1 + LPS**	1.04 ± 0.29	1.26 ± 0.58	n.s.
	**HT5**	0.96 ± 0.24	1.39 ± 0.89	n.s.
	**HT5 + LPS**	1.26 ± 0.42	1 ± 0.61	n.s.
	**HT10**	1.35 ± 0.4	1.04 ± 0.28	n.s.
	**HT10 + LPS**	1.08 ± 0.6	1.3 ± 0.7	n.s.
Repeated measures ANOVA		n.s.	n.s.	

Results are expressed as mean ± standard deviation. Statistical analysis is discussed in detail in the text. n, number of subjects; Untreated, cells that received no treatment; ETOH, cells treated only with vehicle (ethanol); LPS, cells treated with 1 μg/mL lipopolysaccharide; OLE1, cells treated with 1 μM oleuropein; OLE1 + LPS, cells pre-treated with 1 μM oleuropein, then incubated with 1 μg/mL LPS; OLE5, cells treated with 5 μM oleuropein; OLE5 + LPS, cells pre-treated with 5 μM oleuropein, then incubated with 1 μg/mL LPS; OLE10, cells treated with 10 μM oleuropein; OLE10 + LPS, cells pre-treated with 10 μM oleuropein, then incubated with 1 μg/mL LPS; HT1, cells treated with 1 μM hydroxytyrosol; HT1 + LPS, cells pre-treated with 1 μM hydroxytyrosol, then incubated with 1 μg/mL LPS; HT5, cells treated with 5 μM hydroxytyrosol; HT5 + LPS, cells pre-treated with 5 μM hydroxytyrosol, then incubated with 1 μg/mL LPS; HT10, cells treated with 10 μM hydroxytyrosol; HT10 + LPS, cells pre-treated with 10 μM hydroxytyrosol, then incubated with 1 μg/mL LPS; n.s., non-significant.

## Data Availability

Data will be provided upon reasonable request.

## Supplementary Materials

The supporting information can be downloaded at: https://www.mdpi.com/article/10.3390/ijms241311029/s1.

## References

[1] Ferrucci L., Fabbri E. (2018). Inflammageing: Chronic inflammation in ageing, cardiovascular disease, and frailty. Nat. Rev. Cardiol..

[2] Tizazu A.M., Mengist H.M., Demeke G. (2022). Aging, inflammaging and immunosenescence as risk factors of severe COVID-19. Immun. Ageing.

[3] Witkowski J.M., Bryl E., Fulop T. (2021). The role of inflammaging in the development of chronic diseases of older people. Human Aging.

[4] Fülöp T., Larbi A., Pawelec G. (2013). Human T Cell Aging and the Impact of Persistent Viral Infections. Front. Immunol..

[5] Batista M.A., Calvo-Fortes F., Silveira-Nunes G., Camatta G.C., Speziali E., Turroni S., Teixeira-Carvalho A., Martins-Filho O.A., Neretti N., Maioli T.U. (2020). Inflammaging in Endemic Areas for Infectious Diseases. Front. Immunol..

[6] Wagner A., Weinberger B. (2020). Vaccines to Prevent Infectious Diseases in the Older Population: Immunological Challenges and Future Perspectives. Front. Immunol..

[7] Zuo L., Prather E.R., Stetskiv M., Garrison D.E., Meade J.R., Peace T.I., Zhou T. (2019). Inflammaging and oxidative stress in human diseases: From molecular mechanisms to novel treatments. Int. J. Mol. Sci..

[8] Santoro A., Bientinesi E., Monti D. (2021). Immunosenescence and inflammaging in the aging process: Age-related diseases or longevity?. Ageing Res. Rev..

[9] Bektas A., Schurman S.H., Sen R., Ferrucci L. (2018). Aging, inflammation and the environment. Exp. Gerontol..

[10] Kennedy B.K., Berger S.L., Brunet A., Campisi J., Cuervo A.M., Epel E.S., Franceschi C., Lithgow G.J., Morimoto R.I., Pessin J.E. (2014). Geroscience: Linking Aging to Chronic Disease. Cell.

[11] Freund A., Orjalo A.V., Desprez P.-Y., Campisi J. (2010). Inflammatory networks during cellular senescence: Causes and consequences. Trends Mol. Med..

[12] Crooke S.N., Ovsyannikova I.G., Poland G.A., Kennedy R.B. (2019). Immunosenescence and human vaccine immune responses. Immun. Ageing.

[13] Aiello A., Farzaneh F., Candore G., Caruso C., Davinelli S., Gambino C.M., Ligotti M.E., Zareian N., Accardi G. (2019). Immunosenescence and Its Hallmarks: How to Oppose Aging Strategically? A Review of Potential Options for Therapeutic Intervention. Front. Immunol..

[14] Teissier T., Boulanger E., Cox L.S. (2022). Interconnections between Inflammageing and Immunosenescence during Ageing. Cells.

[15] Fulop T., Larbi A., Pawelec G., Khalil A., Cohen A.A., Hirokawa K., Witkowski J.M., Franceschi C. (2021). Immunology of Aging: The Birth of Inflammaging. Clin. Rev. Allergy Immunol..

[16] Fulop T., Larbi A., Dupuis G., Le Page A., Frost E.H., Cohen A.A., Witkowski J.M., Franceschi C. (2018). Immunosenescence and Inflamm-Aging as Two Sides of the Same Coin: Friends or Foes?. Front. Immunol..

[17] Bleve A., Motta F., Durante B., Pandolfo C., Selmi C., Sica A. (2022). Immunosenescence, Inflammaging, and Frailty: Role of Myeloid Cells in Age-Related Diseases. Clin. Rev. Allergy Immunol..

[18] Pawelec G. (2018). Age and immunity: What is “immunosenescence”?. Exp. Gerontol..

[19] Mittelbrunn M., Kroemer G. (2021). Hallmarks of T cell aging. Nat. Immunol..

[20] Guimarães G.R., Almeida P.P., de Oliveira Santos L.D.O., Rodrigues L.P., de Carvalho J.L., Boroni M. (2021). Hallmarks of Aging in Macrophages: Consequences to Skin Inflammaging. Cells.

[21] de Mol J., Kuiper J., Tsiantoulas D., Foks A.C. (2021). The Dynamics of B Cell Aging in Health and Disease. Front. Immunol..

[22] Rocamora-Reverte L., Melzer F.L., Würzner R., Weinberger B. (2020). The Complex Role of Regulatory T Cells in Immunity and Aging. Front. Immunol..

[23] Jagger A., Shimojima Y., Goronzy J.J., Weyand C.M. (2014). Regulatory T cells and the immune aging process: A mini-review. Gerontology.

[24] Heath J.J., Grant M.D. (2020). The Immune Response against Human Cytomegalovirus Links Cellular to Systemic Senescence. Cells.

[25] Sharifi-Rad M., Anil Kumar N.V., Zucca P., Varoni E.M., Dini L., Panzarini E., Rajkovic J., Tsouh Fokou P.V., Azzini E., Peluso I. (2020). Lifestyle, Oxidative Stress, and Antioxidants: Back and Forth in the Pathophysiology of Chronic Diseases. Front. Physiol..

[26] Liguori I., Russo G., Curcio F., Bulli G., Aran L., DELLA-Morte D., Gargiulo G., Testa G., Cacciatore F., Bonaduce D. (2018). Oxidative stress, aging, and diseases. Clin. Interv. Aging.

[27] Serrano-López J., Martín-Antonio B. (2021). Inflammaging, an Imbalanced Immune Response That Needs to Be Restored for Cancer Prevention and Treatment in the Elderly. Cells.

[28] Govindasamy V., Rajendran A., Lee Z., Ooi G., Then K., Then K., Gayathri M., Das A.K., Cheong S. (2021). The potential role of mesenchymal stem cells in modulating antiageing process. Cell Biol. Int..

[29] Birch J., Gil J. (2020). Senescence and the SASP: Many therapeutic avenues. Genes Dev..

[30] Xia S., Zhang X., Zheng S., Khanabdali R., Kalionis B., Wu J., Wan W., Tai X. (2016). An Update on Inflamm-Aging: Mechanisms, Prevention, and Treatment. J. Immunol. Res..

[31] Shields H.J., Traa A., Van Raamsdonk J.M. (2021). Beneficial and Detrimental Effects of Reactive Oxygen Species on Lifespan: A Comprehensive Review of Comparative and Experimental Studies. Front. Cell Dev. Biol..

[32] Untersmayr E., Brandt A., Koidl L., Bergheim I. (2022). The Intestinal Barrier Dysfunction as Driving Factor of Inflammaging. Nutrients.

[33] Thevaranjan N., Puchta A., Schulz C., Naidoo A., Szamosi J., Verschoor C.P., Loukov D., Schenck L.P., Jury J., Foley K.P. (2018). Age-Associated Microbial Dysbiosis Promotes Intestinal Permeability, Systemic Inflammation, and Macrophage Dysfunction. Cell Host Microbe.

[34] Kim K.-A., Jeong J.-J., Yoo S.-Y., Kim D.-H. (2016). Gut microbiota lipopolysaccharide accelerates inflamm-aging in mice. BMC Microbiol..

[35] Gambino C.M., Accardi G., Aiello A., Candore G., Guccione G.D., Mirisola M., Procopio A., Taormina G., Caruso C. (2018). Effect of Extra Virgin Olive Oil and Table Olives on the ImmuneInflammatory Responses: Potential Clinical Applications. Endocrine, Metab. Immune Disord. Drug Targets.

[36] Stromsnes K., Correas A.G., Lehmann J., Gambini J., Olaso-Gonzalez G. (2021). Anti-Inflammatory Properties of Diet: Role in Healthy Aging. Biomedicines.

[37] Losito I., Abbattista R., De Ceglie C., Castellaneta A., Calvano C.D., Cataldi T.R. (2021). Bioactive Secoiridoids in Italian Extra-Virgin Olive Oils: Impact of Olive Plant Cultivars, Cultivation Regions and Processing. Molecules.

[38] Santangelo C., Varì R., Scazzocchio B., De Sancti P., Giovannini C., D‘archivio M., Masella R. (2017). Anti-inflammatory Activity of Extra Virgin Olive Oil Polyphenols: Which Role in the Prevention and Treatment of Immune-Mediated Inflammatory Diseases?. Endocrine, Metab. Immune Disord. Drug Targets.

[39] Tsigalou C., Konstantinidis T., Paraschaki A., Stavropoulou E., Voidarou C., Bezirtzoglou E. (2020). Mediterranean Diet as a Tool to Combat Inflammation and Chronic Diseases. An Overview. Biomedicines.

[40] Capurso C., Bellanti F., Buglio A.L., Vendemiale G. (2019). The Mediterranean Diet Slows Down the Progression of Aging and Helps to Prevent the Onset of Frailty: A Narrative Review. Nutrients.

[41] Zhang J., Zhao A. (2021). Dietary Diversity and Healthy Aging: A Prospective Study. Nutrients.

[42] Dominguez L.J., Di Bella G., Veronese N., Barbagallo M. (2021). Impact of Mediterranean Diet on Chronic Non-Communicable Diseases and Longevity. Nutrients.

[43] Petrella C., Di Certo M.G., Gabanella F., Barbato C., Ceci F.M., Greco A., Ralli M., Polimeni A., Angeloni A., Severini C. (2021). Mediterranean Diet, Brain and Muscle: Olive Polyphenols and Resveratrol Protection in Neurodegenerative and Neuromuscular Disorders. Curr. Med. Chem..

[44] Al-Aubaidy H.A., Dayan A., Deseo M.A., Itsiopoulos C., Jamil D., Hadi N.R., Thomas C.J. (2021). Twelve-Week Mediterranean Diet Intervention Increases Citrus Bioflavonoid Levels and Reduces Inflammation in People with Type 2 Diabetes Mellitus. Nutrients.

[45] Bucciantini M., Leri M., Nardiello P., Casamenti F., Stefani M. (2021). Olive Polyphenols: Antioxidant and Anti-Inflammatory Properties. Antioxidants.

[46] Martinotti S., Bonsignore G., Patrone M., Ranzato E. (2021). Mediterranean Diet Polyphenols: Anthocyanins and Their Implications for Health. Mini-Reviews Med. Chem..

[47] Gorzynik-Debicka M., Przychodzen P., Cappello F., Kuban-Jankowska A., Marino Gammazza A., Knap N., Wozniak M., Gorska-Ponikowska M. (2018). Potential Health Benefits of Olive Oil and Plant Polyphenols. Int. J. Mol. Sci..

[48] Yuan J.-J., Wang C.-Z., Ye J.-Z., Tao R., Zhang Y.-S. (2015). Enzymatic Hydrolysis of Oleuropein from Olea europea (Olive) Leaf Extract and Antioxidant Activities. Molecules.

[49] Qabaha K., Al-Rimawi F., Qasem A., Naser S.A. (2018). Oleuropein Is Responsible for the Major Anti-Inflammatory Effects of Olive Leaf Extract. J. Med. Food.

[50] Marković A.K., Torić J., Barbarić M., Brala C.J. (2019). Hydroxytyrosol, Tyrosol and Derivatives and Their Potential Effects on Human Health. Molecules.

[51] Zorić N., Kopjar N., Rodriguez J.V., Tomić S., Kosalec I. (2021). Protective effects of olive oil phenolics oleuropein and hydroxytyrosol against hydrogen peroxide-induced DNA damage in human peripheral lymphocytes. Acta Pharm..

[52] Pojero F., Aiello A., Gervasi F., Caruso C., Ligotti M.E., Calabrò A., Procopio A., Candore G., Accardi G., Allegra M. (2022). Effects of Oleuropein and Hydroxytyrosol on Inflammatory Mediators: Consequences on Inflammaging. Int. J. Mol. Sci..

[53] Nikou T., Sakavitsi M.E., Kalampokis E., Halabalaki M. (2022). Metabolism and Bioavailability of Olive Bioactive Constituents Based on In Vitro, In Vivo and Human Studies. Nutrients.

[54] Žugčić T., Abdelkebir R., Alcantara C., Collado M.C., Garcia-Perez J.V., Meléndez-Martínez A.J., Režek Jambrak A., Lorenzo J.M., Barba F.J. (2019). From extraction of valuable compounds to health promoting benefits of olive leaves through bioaccessibility, bioavailability and impact on gut microbiota. Trends Food Sci. Technol..

[55] Alemán-Jiménez C., Domínguez-Perles R., Medina S., Prgomet I., López-González I., Simonelli-Muñoz A., Campillo-Cano M., Auñón D., Ferreres F., Gil-Izquierdo A. (2021). Pharmacokinetics and bioavailability of hydroxytyrosol are dependent on the food matrix in humans. Eur. J. Nutr..

[56] de Bock M., Thorstensen E.B., Derraik J.G.B., Henderson H.V., Hofman P.L., Cutfield W.S. (2013). Human absorption and metabolism of oleuropein and hydroxytyrosol ingested as olive (*Olea europaea* L.) leaf extract. Mol. Nutr. Food Res..

[57] Miro-Casas E., Covas M.-I., Farre M., Fito M., Ortuño J., Weinbrenner T., Roset P., de la Torre R. (2003). Hydroxytyrosol Disposition in Humans. Clin. Chem..

[58] Price G., Patel D.A. (2023). Drug Bioavailability.

[59] Vissers M.N., Zock P.L., Roodenburg A.J.C., Leenen R., Katan M.B. (2002). Olive Oil Phenols Are Absorbed in Humans. J. Nutr..

[60] Oliveras-López M.-J., Berná G., Carneiro E.M., de la Serrana H.L.-G., Martín F., López M.C. (2008). An Extra-Virgin Olive Oil Rich in Polyphenolic Compounds Has Antioxidant Effects in Of1 Mice. J. Nutr..

[61] Visioli F., Galli C., Grande S., Colonnelli K., Patelli C., Galli G., Caruso D. (2003). Hydroxytyrosol Excretion Differs between Rats and Humans and Depends on the Vehicle of Administration. J. Nutr..

[62] Tan H.-W., Tuck K.L., Stupans I., Hayball P.J. (2003). Simultaneous determination of oleuropein and hydroxytyrosol in rat plasma using liquid chromatography with fluorescence detection. J. Chromatogr. B.

[63] Bender C., Strassmann S., Golz C. (2023). Oral Bioavailability and Metabolism of Hydroxytyrosol from Food Supplements. Nutrients.

[64] Pastor A., Rodríguez-Morató J., Olesti E., Pujadas M., Pérez-Mañá C., Khymenets O., Fitó M., Covas M.-I., Solá R., Motilva M.-J. (2016). Analysis of free hydroxytyrosol in human plasma following the administration of olive oil. J. Chromatogr. A.

[65] Weinbrenner T., Fitó M., de la Torre R., Saez G.T., Rijken P., Tormos C., Coolen S., Albaladejo M.F., Abanades S., Schroder H. (2004). Olive Oils High in Phenolic Compounds Modulate Oxidative/Antioxidative Status in Men. J. Nutr..

[66] Fuccelli R., Fabiani R., Sepporta M., Rosignoli P. (2015). The hydroxytyrosol-dependent increase of TNF-α in LPS-activated human monocytes is mediated by PGE2 and adenylate cyclase activation. Toxicol. Vitr..

[67] Rosignoli P., Fuccelli R., Fabiani R., Servili M., Morozzi G. (2013). Effect of olive oil phenols on the production of inflammatory mediators in freshly isolated human monocytes. J. Nutr. Biochem..

[68] Weyand C.M., Goronzy J.J. (2016). Aging of the Immune System. Mechanisms and Therapeutic Targets. Ann. Am. Thorac. Soc..

[69] Liu J., Wang Y.-J. (2022). Rejuvenating the Immune System: Insights for Anti-Neurodegeneration Strategies. Neurosci. Bull..

[70] Nousis L., Doulias P.-T., Aligiannis N., Bazios D., Agalias A., Galaris D., Mitakou S. (2005). DNA protecting and genotoxic effects of olive oil related components in cells exposed to hydrogen peroxide. Free. Radic. Res..

[71] Ilavarasi K., Kiruthiga P.V., Pandian S.K., Devi K.P. (2011). Hydroxytyrosol, the phenolic compound of olive oil protects human PBMC against oxidative stress and DNA damage mediated by 2,3,7,8-TCDD. Chemosphere.

[72] Mao X., Xia B., Zheng M., Zhou Z. (2019). Assessment of the anti-inflammatory, analgesic and sedative effects of oleuropein from *Olea europaea* L.. Cell. Mol. Biol..

[73] Cui Y., Gao H., Han S., Yuan R., He J., Zhuo Y., Feng Y.-L., Tang M., Feng J., Yang S. (2021). Oleuropein Attenuates Lipopolysaccharide-Induced Acute Kidney Injury In Vitro and In Vivo by Regulating Toll-Like Receptor 4 Dimerization. Front. Pharmacol..

[74] Ryu S.-J., Choi H.-S., Yoon K.-Y., Lee O.-H., Kim K.-J., Lee B.-Y. (2015). Oleuropein Suppresses LPS-Induced Inflammatory Responses in RAW 264.7 Cell and Zebrafish. J. Agric. Food Chem..

[75] Yousefi Z., Mirsanei Z., Bitaraf F.S., Mahdavi S., Mirzaii M., Jafari R. (2022). Dose-dependent effects of oleuropein administration on regulatory T-cells in patients with rheumatoid arthritis: An in vitro approach. Int. J. Immunopathol. Pharmacol..

[76] Amani F., Farsani M.A., Gholami M., Aghamiri S.M.R., Bakhshandeh M., Mohammadi M.H. (2020). The protective effect of oleuropein against radiation-induced cytotoxicity, apoptosis, and genetic damage in cultured human lymphocytes. Int. J. Radiat. Biol..

[77] Kitsati N., Mantzaris M.D., Galaris D. (2016). Hydroxytyrosol inhibits hydrogen peroxide-induced apoptotic signaling via labile iron chelation. Redox Biol..

[78] Ricelli A., Gionfra F., Percario Z., De Angelis M., Primitivo L., Bonfantini V., Antonioletti R., Bullitta S.M., Saso L., Incerpi S. (2020). Antioxidant and Biological Activities of Hydroxytyrosol and Homovanillic Alcohol Obtained from Olive Mill Wastewaters of Extra-Virgin Olive Oil Production. J. Agric. Food Chem..

[79] Bonura A., Vlah S., Longo A., Bulati M., Melis M.R., Cibella F., Colombo P. (2016). Hydroxytyrosol modulates Par j 1-induced IL-10 production by PBMCs in healthy subjects. Immunobiology.

[80] Fabiani R., De Bartolomeo A., Rosignoli P., Servili M., Montedoro G.F., Morozzi G. (2002). Cancer chemoprevention by hydroxytyrosol isolated from virgin olive oil through G1 cell cycle arrest and apoptosis. Eur. J. Cancer Prev..

[81] Granados-Principal S., Quiles J.L., Ramírez-Tortosa C., Ochoa J., Perez-Lopez P., Pulido-Morán M., Ramirez-Tortosa M. (2012). Squalene ameliorates atherosclerotic lesions through the reduction of CD36 scavenger receptor expression in macrophages. Mol. Nutr. Food Res..

[82] Lin Z., Huang Y., Jiang H., Zhang D., Yang Y., Geng X., Li B. (2021). Functional differences and similarities in activated peripheral blood mononuclear cells by lipopolysaccharide or phytohemagglutinin stimulation between human and cynomolgus monkeys. Ann. Transl. Med..

[83] von Haefen C., Mei W., Menk M., Klemz R., Jones A., Wernecke K.-D., Spies C.D. (2010). Ethanol Changes Gene Expression of Transcription Factors and Cytokine Production of CD4+ T-Cell Subsets in PBMCs Stimulated With LPS. Alcohol. Clin. Exp. Res..

[84] Castilla R., González R., Fouad D., Fraga E., Muntané J. (2004). Dual Effect of Ethanol on Cell Death in Primary Culture of Human and Rat Hepatocytes. Alcohol Alcohol..

[85] Chen H., George I., Sperber K. (1998). Effect of Ethanol on Monocytic Function in Human Immunodeficiency Virus Type 1 Infection. Clin. Diagn. Lab. Immunol..

[86] Nguyen S.T., Nguyen H.T.-L., Truong K.D. (2020). Comparative cytotoxic effects of methanol, ethanol and DMSO on human cancer cell lines. Biomed. Res. Ther..

[87] Kar N., Gupta D., Bellare J. (2021). Ethanol affects fibroblast behavior differentially at low and high doses: A comprehensive, dose-response evaluation. Toxicol. Rep..

[88] Levallois C., Rouahi N., Balmes J.-L., Mani J.-C. (1989). Effects of ethanol in vitro on some parameters of the immune response. Drug Alcohol Depend..

[89] Spinozzi F., Agea E., Florucci G., Gerli R., Muscat C., Belia S., Bertotto A. (1992). Ethanol-induced CD3 and CD2 hyporesponsiveness of peripheral blood T lymphocytes. Immunopharmacol. Immunotoxicol..

[90] Tapani E., Taavitsainen M., Lindros K., Vehmas T., Lehtonen E. (1996). Toxicity of Ethanol in Low Concentrations. Acta Radiol..

[91] Grievink H.W., Luisman T., Kluft C., Moerland M., Malone K.E. (2016). Comparison of Three Isolation Techniques for Human Peripheral Blood Mononuclear Cells: Cell Recovery and Viability, Population Composition, and Cell Functionality. Biopreserv. Biobank..

[92] Golke T., Mucher P., Schmidt P., Radakovics A., Repl M., Hofer P., Perkmann T., Fondi M., Schmetterer K.G., Haslacher H. (2022). Delays during PBMC isolation have a moderate effect on yield, but severly compromise cell viability. Clin. Chem. Lab. Med..

[93] Kleeberger C.A., Lyles R.H., Margolick J.B., Rinaldo C.R., Phair J.P., Giorgi J.V. (1999). Viability and Recovery of Peripheral Blood Mononuclear Cells Cryopreserved for up to 12 Years in a Multicenter Study. Clin. Diagn. Lab. Immunol..

[94] Hope C.M., Huynh D., Wong Y.Y., Oakey H., Perkins G.B., Nguyen T., Binkowski S., Bui M., Choo A.Y.L., Gibson E. (2021). Optimization of Blood Handling and Peripheral Blood Mononuclear Cell Cryopreservation of Low Cell Number Samples. Int. J. Mol. Sci..

[95] Liu J., Liu Z., Wang L., He H., Mu H., Sun W., Zhou Y., Liu Y., Ma W., Zhang W. (2021). Bioactivity-guided isolation of immunomodulatory compounds from the fruits of Ligustrum lucidum. J. Ethnopharmacol..

[96] Jin H., Malek T.R. (2006). Redundant and unique regulation of activated mouse B lymphocytes by IL-4 and IL-21. J. Leukoc. Biol..

[97] Ellmeier W., Jung S., Sunshine M.J., Hatam F., Xu Y., Baltimore D., Mano H., Littman D.R. (2000). Severe B Cell Deficiency in Mice Lacking the Tec Kinase Family Members Tec and Btk. J. Exp. Med..

[98] Dumont N., Aubin E., Proulx D.P., Lemieux R., Bazin R. (2009). Increased secretion of hyperimmune antibodies following lipopolysaccharide stimulation of CD40-activated human B cells in vitro. Immunology.

[99] Parekh V.V., Prasad D.V.R., Banerjee P.P., Joshi B.N., Kumar A., Mishra G.C. (2003). B Cells Activated by Lipopolysaccharide, But Not By Anti-Ig and Anti-CD40 Antibody, Induce Anergy in CD8+ T Cells: Role of TGF-β1. J. Immunol..

[100] Poujol F., Monneret G., Pachot A., Textoris J., Venet F. (2015). Altered T Lymphocyte Proliferation upon Lipopolysaccharide Challenge Ex Vivo. PLoS ONE.

[101] Sueyoshi K., Ledderose C., Shen Y., Lee A.H., Shapiro N.I., Junger W.G. (2019). Lipopolysaccharide suppresses T cells by generating extracellular ATP that impairs their mitochondrial function via P2Y11 receptors. J. Biol. Chem..

[102] Tincati C., Bellistrì G.M., Ancona G., Merlini E., Monforte A.D., Marchetti G. (2012). Role of In Vitro Stimulation with Lipopolysaccharide on T-Cell Activation in HIV-Infected Antiretroviral-Treated Patients. Clin. Dev. Immunol..

[103] Goodier M.R., Londei M. (2000). Lipopolysaccharide Stimulates the Proliferation of Human CD56+CD3− NK Cells: A Regulatory Role of Monocytes and IL-10. J. Immunol..

[104] Brauning A., Rae M., Zhu G., Fulton E., Admasu T.D., Stolzing A., Sharma A. (2022). Aging of the Immune System: Focus on Natural Killer Cells Phenotype and Functions. Cells.

[105] Cooper M.A., Fehniger T.A., Turner S.C., Chen K.S., Ghaheri B.A., Ghayur T., Carson I.W.E., Caligiuri M.A. (2001). Human natural killer cells: A unique innate immunoregulatory role for the CD56^bright^ subset. Blood.

[106] Amand M., Iserentant G., Poli A., Sleiman M., Fievez V., Sanchez I.P., Sauvageot N., Michel T., Aouali N., Janji B. (2017). Human CD56dimCD16dim Cells as an Individualized Natural Killer Cell Subset. Front. Immunol..

[107] Zimmer J. (2020). CD56dimCD16dim Natural Killer (NK) Cells: The Forgotten Population. Hemasphere.

[108] Forconi C.S., Oduor C.I., Oluoch P., Ong’Echa J.M., Münz C., Bailey J.A., Moormann A.M. (2020). A New Hope for CD56negCD16pos NK Cells as Unconventional Cytotoxic Mediators: An Adaptation to Chronic Diseases. Front. Cell. Infect. Microbiol..

[109] Mavilio D., Lombardo G., Benjamin J., Kim D., Follman D., Marcenaro E., O’Shea M.A., Kinter A., Kovacs C., Moretta A. (2005). Characterization of CD56-/CD16+ natural killer (NK) cells: A highly dysfunctional NK subset expanded in HIV-infected viremic individuals. Proc. Natl. Acad. Sci. USA.

[110] Wijaya R.S., Read S.A., Schibeci S., Han S., Azardaryany M.K., van der Poorten D., Lin R., Yuen L., Lam V., Douglas M.W. (2021). Expansion of dysfunctional CD56-CD16+ NK cells in chronic hepatitis B patients. Liver Int..

[111] Vitale M., Della Chiesa M., Carlomagno S., Romagnani C., Thiel A., Moretta L., Moretta A. (2004). The small subset of CD56brightCD16– natural killer cells is selectively responsible for both cell proliferation and interferon-γ production upon interaction with dendritic cells. Eur. J. Immunol..

[112] Chidrawar S.M., Khan N., Chan Y.L.T., Nayak L., Moss P.A. (2006). Ageing is associated with a decline in peripheral blood CD56bright NK cells. Immun. Ageing.

[113] Caligiuri M.A., Murray C., Robertson M.J., Wang E., Cochran K., Cameron C., Schow P., Ross M.E., Klumpp T.R., Soiffer R.J. (1993). Selective modulation of human natural killer cells in vivo after prolonged infusion of low dose recombinant interleukin 2. J. Clin. Investig..

[114] Rodella L., Zamai L., Rezzani R., Artico M., Peri G., Falconi M., Facchini A., Pelusi G., Vitale M. (2001). Interleukin 2 and interleukin 15 differentially predispose natural killer cells to apoptosis mediated by endothelial and tumour cells. Br. J. Haematol..

[115] Knobloch J., Chikosi S.-J., Yanik S., Rupp J., Jungck D., Koch A. (2016). A systemic defect in Toll-like receptor 4 signaling increases lipopolysaccharide-induced suppression of IL-2-dependent T-cell proliferation in COPD. Am. J. Physiol.-Lung Cell. Mol. Physiol..

[116] Minciullo P.L., Catalano A., Mandraffino G., Casciaro M., Crucitti A., Maltese G., Morabito N., Lasco A., Gangemi S., Basile G. (2016). Inflammaging and Anti-Inflammaging: The Role of Cytokines in Extreme Longevity. Arch. Immunol. Ther. Exp..

[117] Mak T.W., Saunders M.E. (2006). Cytokines and Cytokine Receptors. The Immune Response.

[118] Carson W.E., Giri J.G., Lindemann M.J., Linett M.L., Ahdieh M., Paxton R., Anderson D., Eisenmann J., Grabstein K., Caligiuri M.A. (1994). Interleukin (IL) 15 is a novel cytokine that activates human natural killer cells via components of the IL-2 receptor. J. Exp. Med..

[119] Gangemi S., Basile G., Monti D., Merendino R.A., Di Pasquale G., Bisignano U., Nicita-Mauro V., Franceschi C. (2005). Age-Related Modifications in Circulating IL-15 Levels in Humans. Mediat. Inflamm..

[120] Cooley S., Xiao F., Pitt M., Gleason M., McCullar V., Bergemann T.L., McQueen K.L., Guethlein L.A., Parham P., Miller J.S. (2007). A subpopulation of human peripheral blood NK cells that lacks inhibitory receptors for self-MHC is developmentally immature. Blood.

[121] Dubois S., Conlon K.C., Müller J.R., Hsu-Albert J., Beltran N., Bryant B.R., Waldmann T.A. (2017). IL15 Infusion of Cancer Patients Expands the Subpopulation of Cytotoxic CD56bright NK Cells and Increases NK-Cell Cytokine Release Capabilities. Cancer Immunol. Res..

[122] Magrone T., Spagnoletta A., Salvatore R., Magrone M., Dentamaro F., Russo M.A., Difonzo G., Summo C., Caponio F., Jirillo E. (2017). Olive Leaf Extracts Act as Modulators of the Human Immune Response. Endocr. Metab. Immune Disord. Drug Targets.

[123] Narasimhan P.B., Marcovecchio P., Hamers A.A., Hedrick C.C. (2019). Nonclassical Monocytes in Health and Disease. Annu. Rev. Immunol..

[124] Yang J., Zhang L., Yu C., Yang X.-F., Wang H. (2014). Monocyte and macrophage differentiation: Circulation inflammatory monocyte as biomarker for inflammatory diseases. Biomark. Res..

[125] Ammann S., Fuchs S., Martin-Martin L., Castro C.N., Spielberger B., Klemann C., Elling R., Heeg M., Speckmann C., Hainmann I. (2020). Functional flow cytometry of monocytes for routine diagnosis of innate primary immunodeficiencies. J. Allergy Clin. Immunol..

[126] Mukherjee R., Barman P.K., Thatoi P.K., Tripathy R., Das B.K., Ravindran B. (2015). Non-Classical monocytes display inflammatory features: Validation in Sepsis and Systemic Lupus Erythematous. Sci. Rep..

[127] Waller K., James C., De Jong A., Blackmore L., Ma Y., Stagg A., Kelsell D., O’Dwyer M., Hutchins R., Alazawi W. (2019). ADAM17-Mediated Reduction in CD14++CD16+ Monocytes ex vivo and Reduction in Intermediate Monocytes with Immune Paresis in Acute Pancreatitis and Acute Alcoholic Hepatitis. Front. Immunol..

[128] Zhou L., Ge M., Zhang Y., Wu X., Leng M., Gan C., Mou Y., Zhou J., Valencia C.A., Hao Q. (2022). Centenarians Alleviate Inflammaging by Changing the Ratio and Secretory Phenotypes of T Helper 17 and Regulatory T Cells. Front. Pharmacol..

[129] Schildberger A., Rossmanith E., Eichhorn T., Strassl K., Weber V. (2013). Monocytes, Peripheral Blood Mononuclear Cells, and THP-1 Cells Exhibit Different Cytokine Expression Patterns following Stimulation with Lipopolysaccharide. Mediat. Inflamm..

[130] Auffray C., Sieweke M.H., Geissmann F. (2009). Blood Monocytes: Development, Heterogeneity, and Relationship with Dendritic Cells. Annu. Rev. Immunol..

[131] Chaiwut R., Kasinrerk W. (2022). Very low concentration of lipopolysaccharide can induce the production of various cytokines and chemokines in human primary monocytes. BMC Res. Notes.

[132] Agarwal S., Piesco N., Johns L., Riccelli A. (1995). Differential Expression of IL-1β, TNF-α, IL-6, and IL-8 in Human Monocytes in Response to Lipopolysaccharides from Different Microbes. J. Dent. Res..

[133] Miles E.A., Zoubouli P., Calder P.C. (2005). Differential anti-inflammatory effects of phenolic compounds from extra virgin olive oil identified in human whole blood cultures. Nutrition.

[134] Martin M.E., Millan-Linares M.C., Naranjo M.C., Toscano R., Abia R., Muriana F.J.G., Bermudez B., la Paz S.M. (2019). Minor compounds from virgin olive oil attenuate LPS-induced inflammation via visfatin-related gene modulation on primary human monocytes. J. Food Biochem..

[135] Zhang X., Cao J., Zhong L. (2009). Hydroxytyrosol inhibits pro-inflammatory cytokines, iNOS, and COX-2 expression in human monocytic cells. Naunyn-Schmiedeberg’s Arch. Pharmacol..

[136] Bigagli E., Cinci L., Paccosi S., Parenti A., D’Ambrosio M., Luceri C. (2017). Nutritionally relevant concentrations of resveratrol and hydroxytyrosol mitigate oxidative burst of human granulocytes and monocytes and the production of pro-inflammatory mediators in LPS-stimulated RAW 264.7 macrophages. Int. Immunopharmacol..

[137] Yu Y.-B., Zhuang H.-Z., Ji X.-J., Dong L., Duan M.-L. (2020). Hydroxytyrosol suppresses LPS-induced intrahepatic inflammatory responses via inhibition of ERK signaling pathway activation in acute liver injury. Eur. Rev. Med. Pharmacol. Sci..

[138] Corsini E., Vismara L., Lucchi L., Viviani B., Govoni S., Galli C.L., Marinovich M., Racchi M. (2006). High interleukin-10 production is associated with low antibody response to influenza vaccination in the elderly. J. Leukoc. Biol..

[139] Almanan M., Raynor J., Ogunsulire I., Malyshkina A., Mukherjee S., Hummel S.A., Ingram J.T., Saini A., Xie M.M., Alenghat T. (2020). IL-10–producing Tfh cells accumulate with age and link inflammation with age-related immune suppression. Sci. Adv..

[140] Rea I.M., Gibson D.S., McGilligan V., McNerlan S.E., Alexander H.D., Ross O.A. (2018). Age and Age-Related Diseases: Role of Inflammation Triggers and Cytokines. Front. Immunol..

[141] Hirokawa K., Utsuyama M., Hayashi Y., Kitagawa M., Makinodan T., Fulop T. (2013). Slower immune system aging in women versus men in the Japanese population. Immun. Ageing.

[142] Wong K.L., Yeap W.H., Tai J.J.Y., Ong S.M., Dang T.M., Wong S.C. (2012). The three human monocyte subsets: Implications for health and disease. Immunol. Res..

[143] Saraiva M., Vieira P., O’garra A. (2020). Biology and therapeutic potential of interleukin-10. J. Exp. Med..

[144] Serreli G., Deiana M. (2018). Biological Relevance of Extra Virgin Olive Oil Polyphenols Metabolites. Antioxidants.

[145] De Leonardis A., Macciola V., Iacovino S. (2020). Delivery Systems for Hydroxytyrosol Supplementation: State of the Art. Colloids Interfaces.

[146] Nardi M., Brocchini S., Somavarapu S., Procopio A. (2023). Hydroxytyrosol oleate: A promising neuroprotective nanocarrier delivery system of oleuropein and derivatives. Int. J. Pharm..

[147] Huguet-Casquero A., Xu Y., Gainza E., Pedraz J.L., Beloqui A. (2020). Oral delivery of oleuropein-loaded lipid nanocarriers alleviates inflammation and oxidative stress in acute colitis. Int. J. Pharm..

[148] Zygouri P., Athinodorou A.M., Spyrou K., Simos Y.V., Subrati M., Asimakopoulos G., Vasilopoulos K.C., Vezyraki P., Peschos D., Tsamis K. (2023). Oxidized-Multiwalled Carbon Nanotubes as Non-Toxic Nanocarriers for Hydroxytyrosol Delivery in Cells. Nanomaterials.

[149] Nardi M., Bonacci S., Cariati L., Costanzo P., Oliverio M., Sindona G., Procopio A. (2017). Synthesis and antioxidant evaluation of lipophilic oleuropein aglycone derivatives. Food Funct..

[150] Gambacorta A., Tofani D., Bernini R., Migliorini A. (2007). High-Yielding Preparation of a Stable Precursor of Hydroxytyrosol by Total Synthesis and from the Natural Glycoside Oleuropein. J. Agric. Food Chem..

[151] Wang P., Henning S.M., Heber D. (2010). Limitations of MTT and MTS-Based Assays for Measurement of Antiproliferative Activity of Green Tea Polyphenols. PLoS ONE.

[152] Akter S., Addepalli R., Netzel M.E., Tinggi U., Fletcher M.T., Sultanbawa Y., Osborne S.A. (2019). Antioxidant-Rich Extracts of *Terminalia ferdinandiana* Interfere with Estimation of Cell Viability. Antioxidants.

[153] Lemieux J., Jobin C., Simard C., Néron S. (2016). A global look into human T cell subsets before and after cryopreservation using multiparametric flow cytometry and two-dimensional visualization analysis. J. Immunol. Methods.

[154] Hønge B.L., Petersen M.S., Olesen R., Møller B.K., Erikstrup C. (2017). Optimizing recovery of frozen human peripheral blood mononuclear cells for flow cytometry. PLoS ONE.

[155] Li B., Yang C., Jia G., Liu Y., Na Wang N., Yang F., Su R., Shang Y., Han Y. (2022). Comprehensive evaluation of the effects of long-term cryopreservation on peripheral blood mononuclear cells using flow cytometry. BMC Immunol..

