# Supplementary material for: Anti-Inflammatory Effects of Nutritionally Relevant Concentrations of Oleuropein and Hydroxytyrosol on Peripheral Blood Mononuclear Cells: An Age-Related Analysis
Source: Int J Mol Sci. 2023 Jul 3;24(13):11029. doi: 10.3390/ijms241311029 (PMC10341641; doi:10.3390/ijms241311029)
Supplement: Supplementary file 1 [file ijms-24-11029-s001.zip › ijms-2457241-supplementary.pdf]

SUPPLEMENTARY TABLES

Table S1. Antibodies used in this study

Antigen	Manufacturer	Clone	Flurochrome	Isotype
CD3	Beckman Coulter	UCHT1	FITC	IgG1 Mouse
CD4	Beckman Coulter	SFCI12T4D11	ECD	IgG1 Mouse
CD8	Beckman Coulter	B9.11	PE	IgG1 Mouse
CD19	Beckman Coulter	J3-119	PC5.5	IgG1 Mouse
CD16	Beckman Coulter	3G8	APC-A750	IgG1 Mouse
CD16	Beckman Coulter	3G8	PB	IgG1 Mouse
CD56	Beckman Coulter	N901	PC7	IgG1 Mouse
CD14	Beckman Coulter	RMO52	APC-A700	IgG2a Mouse

All the listed antigens are human. FITC, Fluorescein Isothiocyanate; ECD, R- Phycoerythrin-Texas Red®-; PE, R-Phycoerytrhin; PC5.5, R-Phycoerythrin-Cyanin 5.5; PE, R-Phycoerythrin; APC-A750, Allophycocyanin-Alexa Fluor 750; PB, Pacific Blue; PC7, R-Phycoerythrin-Cyanin 7; APC-A700, Allophycocyanin-Alexa Fluor 700. Beckman Coulter, Brea, California, USA.

Table S2. Characteristics of the flow cytometer used in this study.

Model (manufacturer)	Lasers Detector	Filters
Navios (Beckman Coulter) 10 colors, 3 lasers (5+3+2 configuration)	Blue Solid State Diode: 488nm, 22mW laser output	Forward Scatter: 488/10<br>Blue Laser: 525/40, 575/30, 620/30, 675/20, 695/30, 755LP
	Red Solid State Diode: 638nm, 25mW laser output	Red Laser: 660/20, 725/20, 755 LP
	Violet Solid State Diode: 405nm, 40mW laser output	Violet Laser: 450/50, 550/40

Beckman Coulter, Brea, California, USA.

Table S3. p values for pairwise comparisons of the effects of OLE and HT on IL-8 release in the extracellular medium by PBMCs of 12 donors (6 Adult and 6 Senior) in the presence/absence of LPS

	UNTREATED	ETOH	LPS	OLE1	OLE1 + LPS	OLE5	OLE5 + LPS	OLE10	OLE10 + LPS	HT1	HT1 + LPS	HT5	HT5 + LPS	HT10	HT10 + LPS
UNTREATED	-	n.s.	0.02	n.s.	0.027	n.s.	0.019	n.s.	0.037	n.s	0.02	n.s	0.022	n.s.	n.s.
ETOH	n.s.	-	0.002	n.s.	0.001	n.s.	0.001	n.s.	0.005	n.s	0.002	n.s	0.001	n.s.	0.012
LPS	0.02	0.002	-	0.013	n.s	<0.0005	n.s.	<0.0005	n.s.	0.007	n.s.	0.003	n.s.	0.001	n.s.
OLE1	n.s.	n.s.	0.013	-	0.010	n.s.	0.014	n.s.	0.036	n.s.	0.015	n.s.	0.015	n.s.	n.s.
OLE1 + LPS	0.027	0.001	n.s.	0.01	-	<0.0005	n.s.	<0.0005	n.s.	0.005	n.s.	0.003	n.s.	0.001	n.s.
OLE5	n.s.	n.s.	<0.0005	n.s.	<0.0005	-	<0.0005	n.s.	0.001	n.s.	<0.0005	n.s.	<0.0005	n.s.	0.001
OLE5 + LPS	0.019	0.001	n.s.	0.014	n.s.	<0.0005	-	<0.0005	n.s.	0.007	n.s.	0.004	n.s.	0.001	n.s.
OLE10	n.s.	n.s.	<0.0005	n.s.	<0.0005	n.s.	<0.0005	-	0.001	n.s.	<0.0005	n.s.	<0.0005	n.s.	0.002
OLE10 + LPS	0.037	0.005	n.s.	0.036	n.s.	0.001	n.s.	0.001	-	0.02	n.s.	0.011	n.s.	0.003	n.s.
HT1	n.s.	n.s.	0.007	n.s.	0.005	n.s.	0.007	n.s.	0.02	-	0.008	n.s.	0.007	n.s.	0.042
HT1 + LPS	0.02	0.002	n.s.	0.015	n.s.	<0.0005	n.s.	<0.0005	n.s.	0.008	-	0.004	n.s.	0.001	n.s.
HT5	n.s.	n.s.	0.003	n.s.	0.003	n.s.	0.004	n.s.	0.011	n.s.	0.004	-	0.004	n.s.	0.016
HT5 + LPS	0.022	0.001	n.s.	0.015	n.s.	<0.0005	n.s.	<0.0005	n.s.	0.007	n.s.	0.004	-	0.001	n.s.
HT10	n.s.	n.s.	0.001	n.s.	0.001	n.s.	0.001	n.s.	0.003	n.s.	0.001	n.s.	0.001	-	0.004
HT10 + LPS	n.s.	0.012	n.s.	n.s.	n.s.	0.001	n.s.	0.002	n.s.	0.042	n.s.	0.016	n.s.	0.004	-

Each cell indicates the statistical significance of the considered pairwise comparison. n.s., non-significant; -, no pairwise comparison. UNTREATED, cells that received no treatment; ETOH, cells treated only with vehicle (ethanol); LPS, cells treated with 1 μg/mL lypopolysaccharide; OLE1, cells treated with 1 μM oleuropein; OLE1 + LPS, cells pre-treated with 1 μM oleuropein, then incubated with 1 μg/mL LPS; OLE5, cells treated with 5 μM oleuropein; OLE5 + LPS, cells pre-treated with 5 μM oleuropein, then incubated with 1 μg/mL LPS; OLE10, cells treated with 10 μM oleuropein; OLE10 + LPS, cells pre-treated with 10 μM oleuropein, then incubated with 1 μg/mL LPS; HT1, cells treated with 1 μM hydroxytyrosol; HT1 + LPS, cells pre-treated with 1 μM hydroxytyrosol, then incubated with 1 μg/mL LPS; HT5, cells treated with 5 μM hydroxytyrosol; HT5 + LPS, cells pre-treated with 5 μM hydroxytyrosol, then incubated with 1 μg/mL LPS; HT10, cells treated with 10 μM hydroxytyrosol; HT10 + LPS, cells pre-treated with 10 μM hydroxytyrosol, then incubated with 1 μg/mL LPS.

Table S4. p values for pairwise comparisons of the effects of OLE and HT on IL-8 release in the extracellular medium by PBMCs of the Adult group (6 subjects) in the presence/absence of LPS

	UNTREATED	ETOH	LPS	OLE1	OLE1 + LPS	OLE5	OLE5 + LPS	OLE10	OLE10 + LPS	HT1	HT1 + LPS	HT5	HT5 + LPS	HT10	HT10 + LPS
UNTREATED	-	n.s.	n.s.	n.s.	n.s.	n.s.	n.s.	n.s.	n.s.	n.s.	n.s.	n.s.	n.s.	n.s.	n.s.
ETOH	n.s.	-	n.s.	n.s.	n.s.	n.s.	n.s.	n.s.	n.s.	n.s.	n.s.	n.s.	n.s.	n.s.	n.s.
LPS	n.s.	n.s.	-	n.s.	n.s.	0.013	n.s.	0.002	n.s.	n.s.	n.s.	n.s.	n.s.	0.005	n.s.
OLE1	n.s.	n.s.	n.s.	-	n.s.	n.s.	n.s.	n.s.	n.s.	n.s.	n.s.	n.s.	n.s.	n.s.	n.s.
OLE1 + LPS	n.s.	n.s.	n.s.	n.s.	-	0.014	n.s.	0.002	n.s.	n.s.	n.s.	n.s.	n.s.	0.006	n.s.
OLE5	n.s.	n.s.	0.013	n.s.	0.014	-	0.017	n.s.	0.013	n.s.	0.018	n.s.	0.018	n.s.	0.012
OLE5 + LPS	n.s.	n.s.	n.s.	n.s.	n.s.	0.017	-	0.002	n.s.	n.s.	n.s.	n.s.	n.s.	0.008	n.s.
OLE10	n.s.	n.s.	0.002	n.s.	0.002	n.s.	0.002	-	0.004	n.s.	0.003	n.s.	0.002	n.s.	0.003
OLE10 + LPS	n.s.	n.s.	n.s.	n.s.	n.s.	0.013	n.s.	0.004	-	n.s.	n.s.	n.s.	n.s.	0.006	n.s.
HT1	n.s.	n.s.	n.s.	n.s.	n.s.	n.s.	n.s.	n.s.	n.s.	-	n.s.	n.s.	n.s.	n.s.	n.s.
HT1 + LPS	n.s.	n.s.	n.s.	n.s.	n.s.	0.018	n.s.	0.003	n.s.	n.s.	-	n.s.	n.s.	0.007	n.s.
HT5	n.s.	n.s.	n.s.	n.s.	n.s.	n.s.	n.s.	n.s.	n.s.	n.s.	n.s.	-	n.s.	n.s.	n.s.
HT5 + LPS	n.s.	n.s.	n.s.	n.s.	n.s.	0.018	n.s.	0.002	n.s.	n.s.	n.s.	n.s.	-	0.007	n.s.
HT10	n.s.	n.s.	0.005	n.s.	0.006	n.s.	0.008	n.s.	0.006	n.s.	0.007	n.s.	0.007	-	0.005
HT10 + LPS	n.s.	n.s.	n.s.	n.s.	n.s.	0.012	n.s.	0.003	n.s.	n.s.	n.s.	n.s.	n.s.	0.005	-

Each cell indicates the statistical significance of the considered pairwise comparison. n.s., non-significant; -, no pairwise comparison. UNTREATED, cells that received no treatment; ETOH, cells treated only with vehicle (ethanol); LPS, cells treated with 1 µg/mL lypopolysaccharide; OLE1, cells treated with 1 µM oleuropein; OLE1 + LPS, cells pre-treated with 1 µM oleuropein, then incubated with 1 µg/mL LPS; OLE5, cells treated with 5 µM oleuropein; OLE5 + LPS, cells pre-treated with 5 µM oleuropein, then incubated with 1 µg/mL LPS; OLE10, cells treated with 10 µM oleuropein; OLE10 + LPS, cells pre-treated with 10 µM oleuropein, then incubated with 1 µg/mL LPS; HT1, cells treated with 1 µM hydroxytyrosol; HT1 + LPS, cells pre-treated with 1 µM hydroxytyrosol, then incubated with 1 µg/mL LPS; HT5, cells treated with 5 µM hydroxytyrosol; HT5 + LPS, cells pre-treated with 5 µM hydroxytyrosol, then incubated with 1 µg/mL LPS; HT10, cells treated with 10 µM hydroxytyrosol; HT10 + LPS, cells pre-treated with 10 µM hydroxytyrosol, then incubated with 1 µg/mL LPS.

FIGURE S1

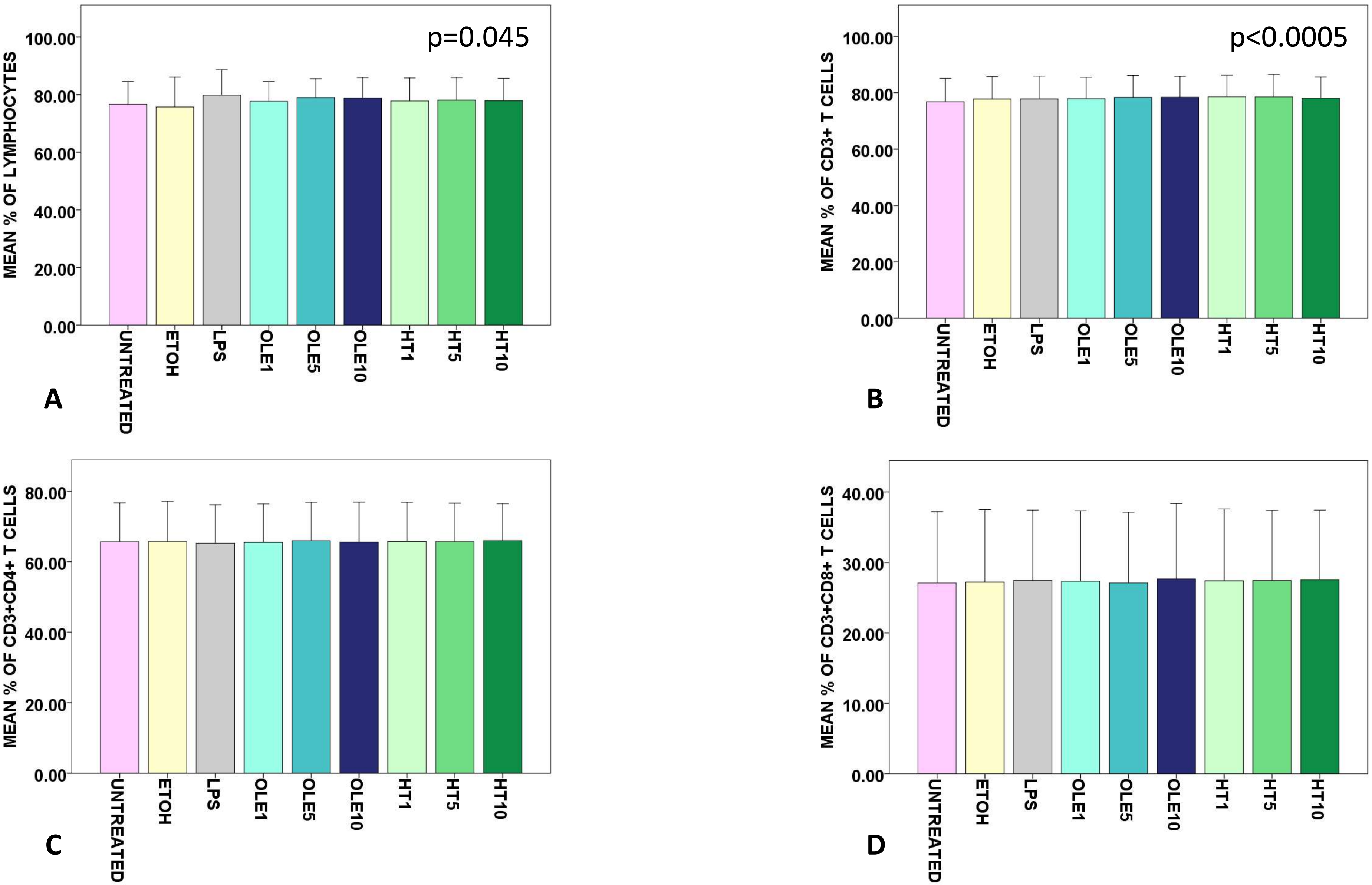
A
MEAN % OF LYMPHOCYTES
p=0.045
0.00
20.00
40.00
60.00
80.00
100.00
UNTREATED
ETOH
LPS
OLE1
OLE5
OLE10
HT1
HT5
HT10
B
MEAN % OF CD3+ T CELLS
p<0.0005
C
MEAN % OF CD3+CD4+ T CELLS
D
MEAN % OF CD3+CD8+ T CELLS
10.00
30.00
40.00

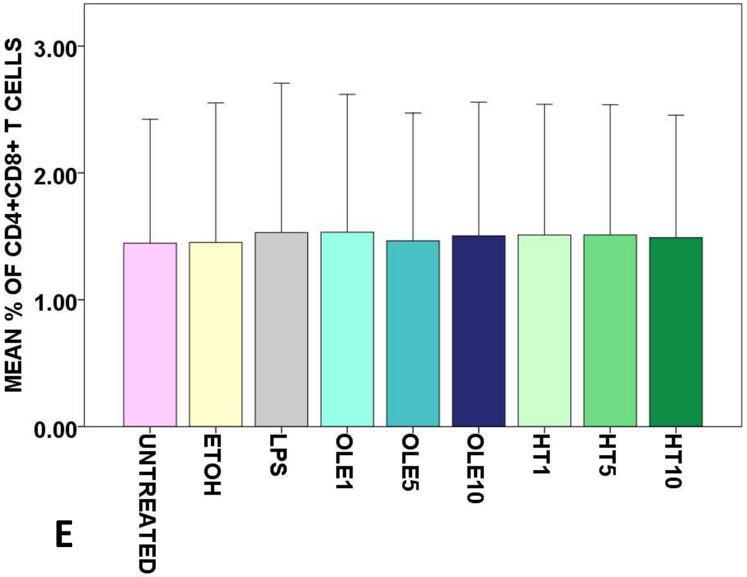
MEAN % OF CD4+CD8+ T CELLS
0.00
1.00
2.00
3.00
UNTREATED
ETOH
LPS
OLE1
OLE5
OLE10
HT1
HT5
HT10

E

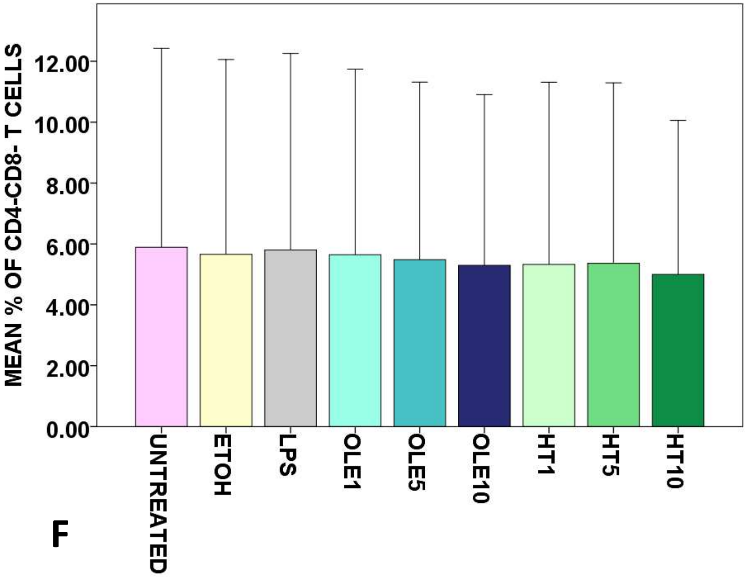
MEAN % OF CD4-CD8- T CELLS
0.00
2.00
4.00
6.00
8.00
10.00
12.00
UNTREATED
ETOH
LPS
OLE1
OLE5
OLE10
HT1
HT5
HT10

F

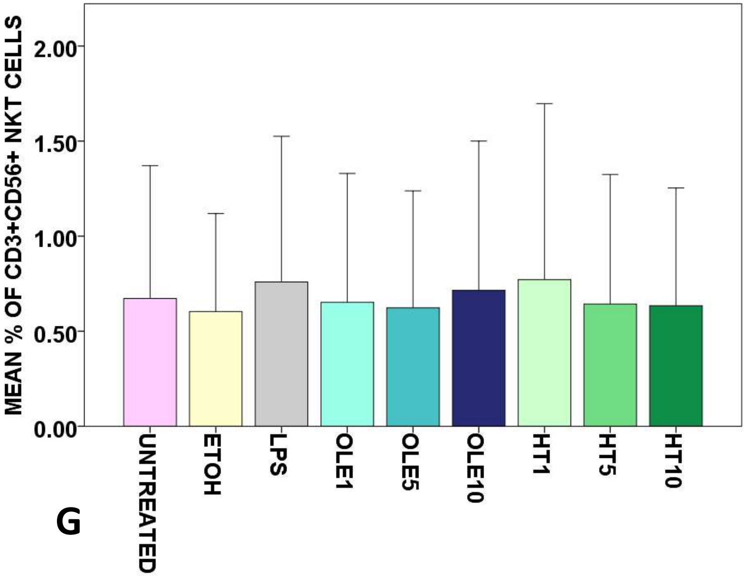
MEAN % OF CD3+CD56+ NKT CELLS
0.00
0.50
1.00
1.50
2.00
UNTREATED
ETOH
LPS
OLE1
OLE5
OLE10
HT1
HT5
HT10

G

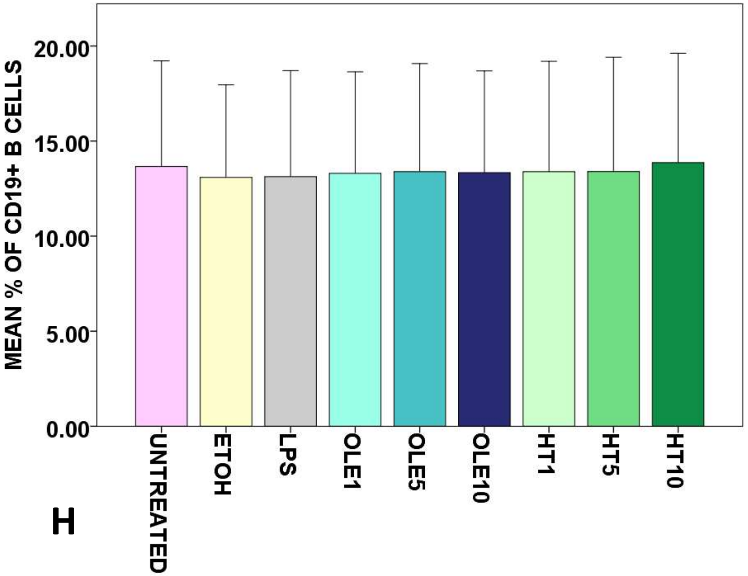
MEAN % OF CD19+ B CELLS
0.00
5.00
10.00
15.00
20.00
UNTREATED
ETOH
LPS
OLE1
OLE5
OLE10
HT1
HT5
HT10

H

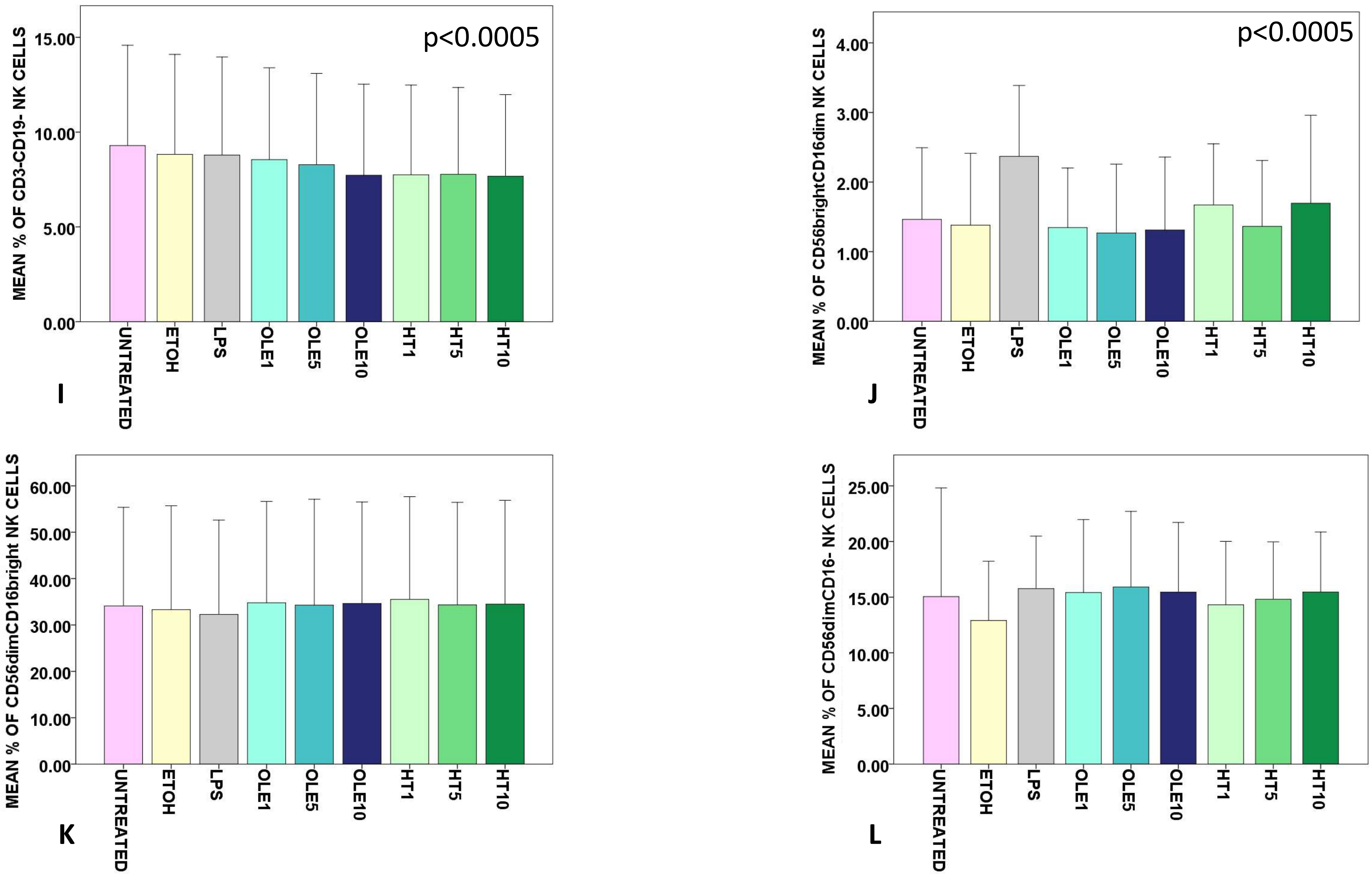

p<0.0005
MEAN % OF CD3-CD19- NK CELLS
15.00
10.00
5.00
0.00
UNTREATED
ETOH
LPS
OLE1
OLE5
OLE10
HT1
HT5
HT10
I
p<0.0005
MEAN % OF CD56brightCD16dim NK CELLS
4.00
3.00
2.00
1.00
0.00
UNTREATED
ETOH
LPS
OLE1
OLE5
OLE10
HT1
HT5
HT10
J
MEAN % OF CD56dimCD16bright NK CELLS
60.00
50.00
40.00
30.00
20.00
10.00
0.00
UNTREATED
ETOH
LPS
OLE1
OLE5
OLE10
HT1
HT5
HT10
K
MEAN % OF CD56dimCD16- NK CELLS
25.00
20.00
15.00
10.00
5.00
0.00
UNTREATED
ETOH
LPS
OLE1
OLE5
OLE10
HT1
HT5
HT10
L

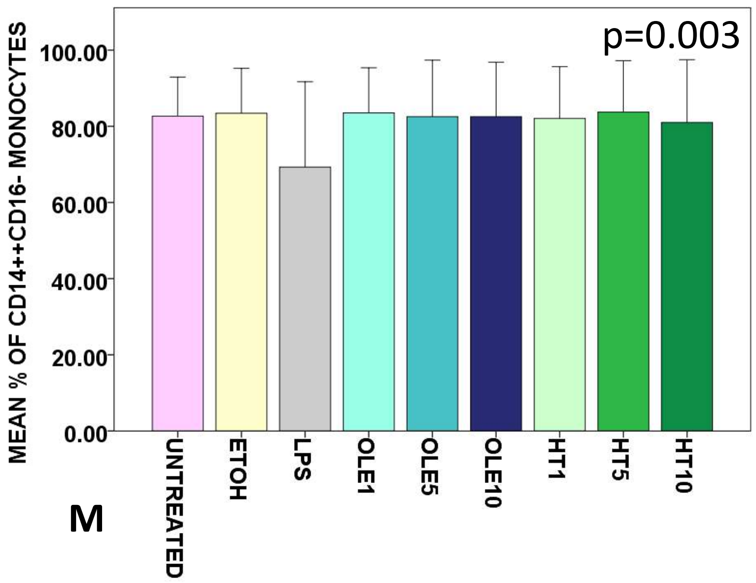
p=0.003
MEAN % OF CD14++CD16- MONOCYTES
100.00
80.00
60.00
40.00
20.00
0.00
UNTREATED
ETOH
LPS
OLE1
OLE5
OLE10
HT1
HT5
HT10

M

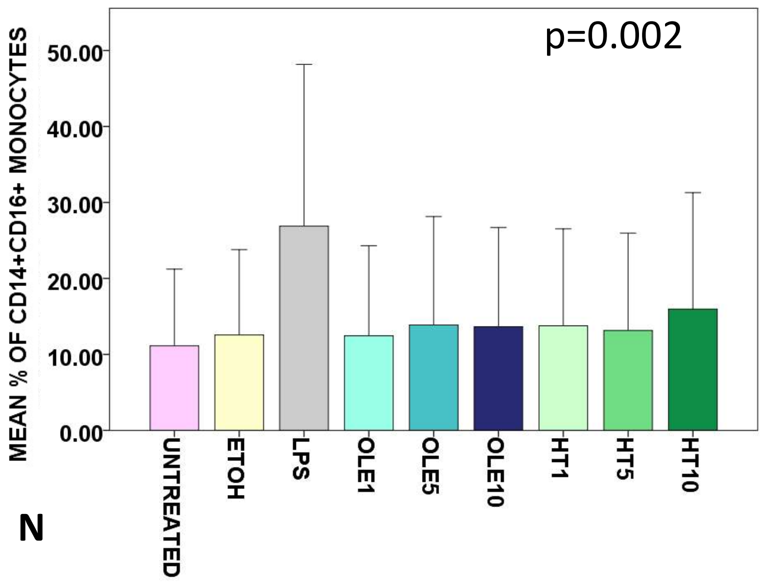
p=0.002
MEAN % OF CD14+CD16+ MONOCYTES
50.00
40.00
30.00
20.00
10.00
0.00
UNTREATED
ETOH
LPS
OLE1
OLE5
OLE10
HT1
HT5
HT10

N

FIGURE S1. **Effects of OLE and HT on the frequency of lymphocyte and monocyte subsets as assayed by flow cytometry on PBMCs.** Data were obtained for 10 donors (5 Adult and 5 Senior). Each diagram represent PBMC population/subset for which only values that did not reach statistical significance at pairwise comparison, and non-significant data were obtained. The mean percentages of the studied lymphocyte or monocyte subsets recorded for each experimental condition are showed as bars, whereas error bars represent one standard deviation. Statistical significance as detected at repeated measures ANOVA is reported in the corresponding graphics. Panels show data as follows: A, lymphocytes; B, T cells; C, CD4+ T cells; D, CD8+ T cells; E, CD4+CD8+ T cells; F, CD4−CD8− T cells; G, CD3+CD56+ NKT cells; H, B cells; I, NK cells; J, CD56brightCD16dim NK cells; K, CD56dimCD16bright NK cells; L, CD56dimCD16− NK cells; M, CD14++CD16− classical monocytes; N, CD14+CD16+ non-classical monocytes. %, percentage; UNTREATED, cells that received no treatment; ETOH, cells treated only with vehicle (ethanol); LPS, cells treated with 1 µg/mL lypopolysaccharide; OLE1, cells treated with 1 µM oleuropein; OLE5, cells treated with 5 µM oleuropein; OLE10, cells treated with 10 µM oleuropein; HT1, cells treated with 1 µM hydroxytyrosol; HT5, cells treated with 5 µM hydroxytyrosol; HT10, cells treated with 10 µM hydroxytyrosol. SD, standard deviation.

FIGURE S2

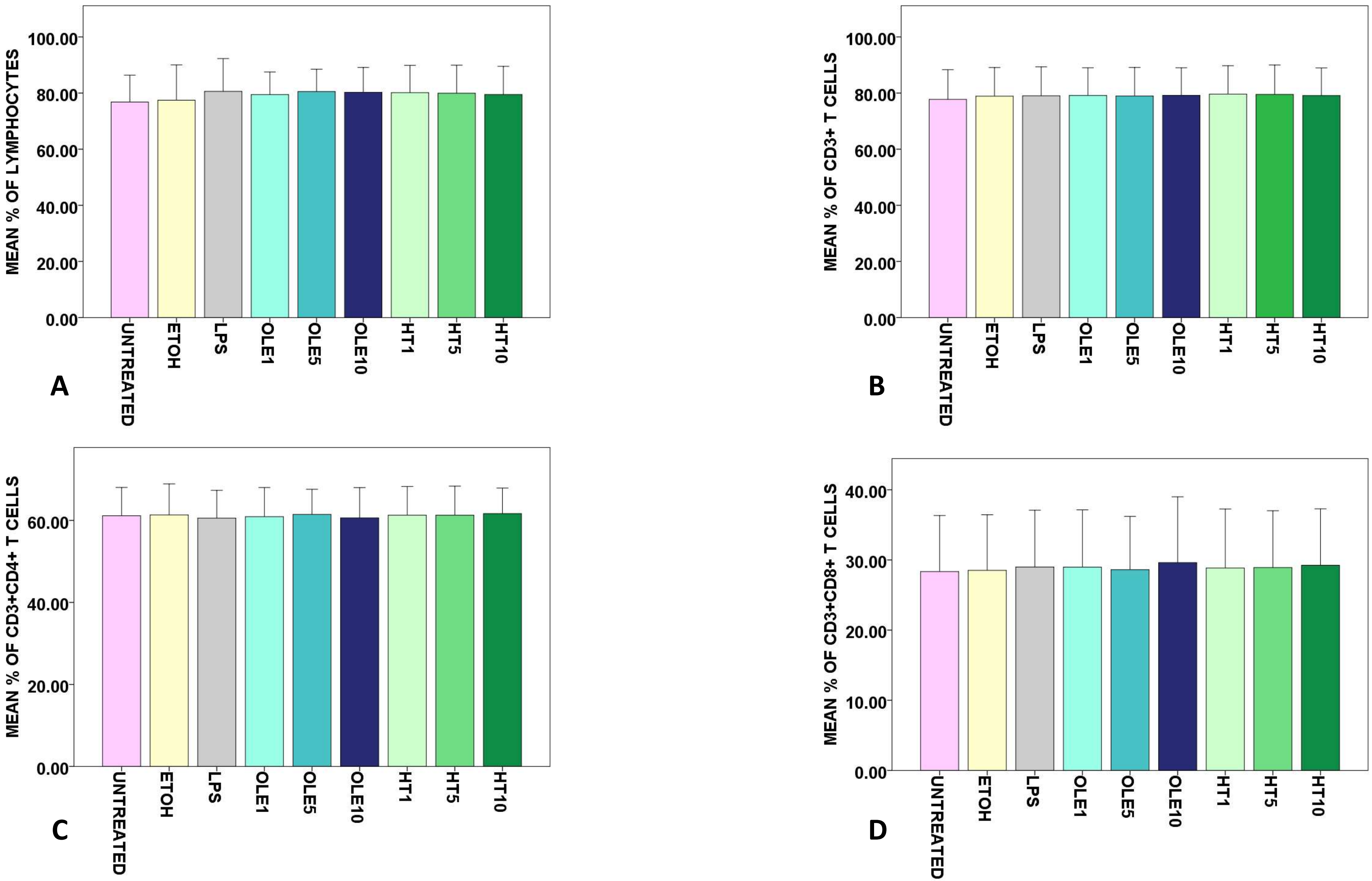

A
MEAN % OF LYMPHOCYTES
0.00
20.00
40.00
60.00
80.00
100.00
UNTREATED
ETOH
LPS
OLE1
OLE5
OLE10
HT1
HT5
HT10
B
MEAN % OF CD3+ T CELLS
0.00
20.00
40.00
60.00
80.00
100.00
UNTREATED
ETOH
LPS
OLE1
OLE5
OLE10
HT1
HT5
HT10
C
MEAN % OF CD3+CD4+ T CELLS
0.00
20.00
40.00
60.00
UNTREATED
ETOH
LPS
OLE1
OLE5
OLE10
HT1
HT5
HT10
D
MEAN % OF CD3+CD8+ T CELLS
0.00
10.00
20.00
30.00
40.00
UNTREATED
ETOH
LPS
OLE1
OLE5
OLE10
HT1
HT5
HT10

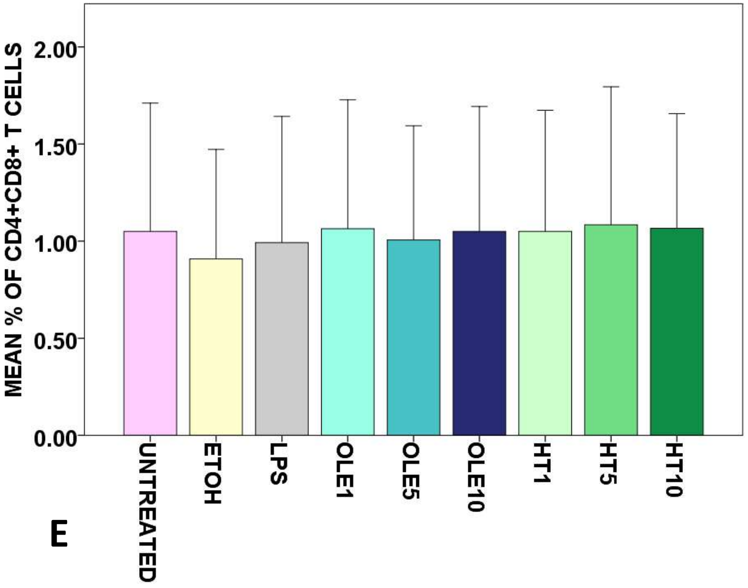
MEAN % OF CD4+CD8+ T CELLS
2.00
1.50
1.00
0.50
0.00
UNTREATED
ETOH
LPS
OLE1
OLE5
OLE10
HT1
HT5
HT10

E

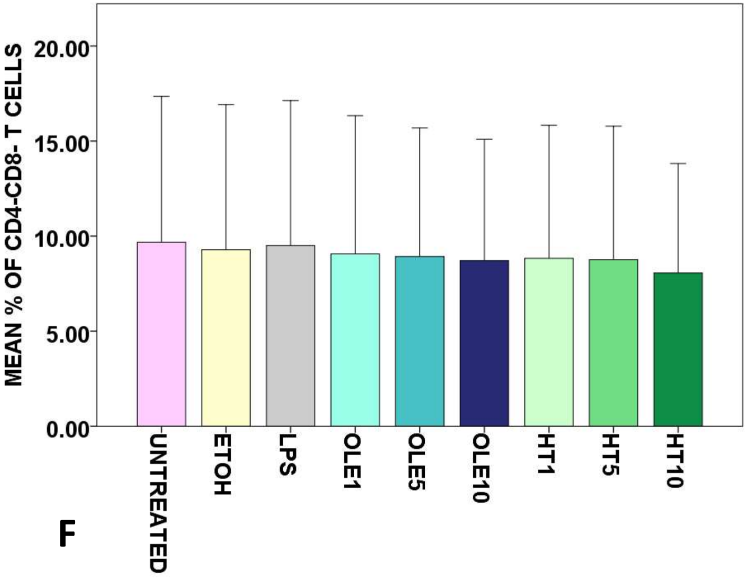
MEAN % OF CD4-CD8- T CELLS
20.00
15.00
10.00
5.00
0.00
UNTREATED
ETOH
LPS
OLE1
OLE5
OLE10
HT1
HT5
HT10

F

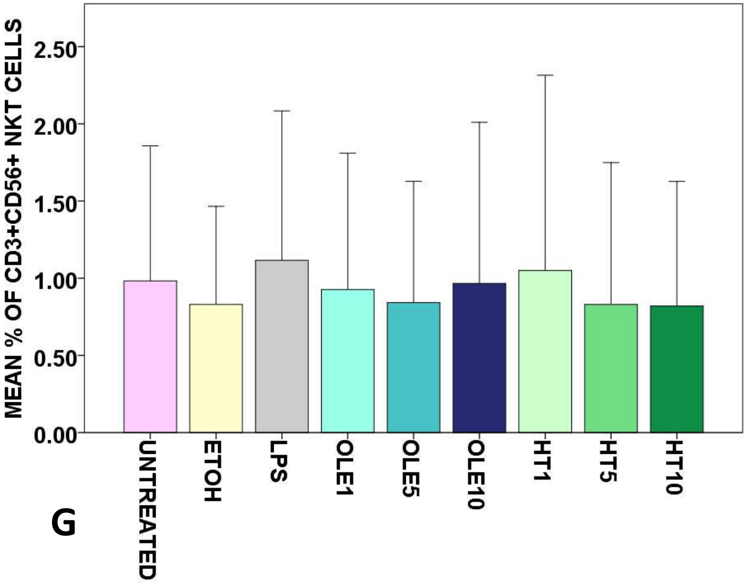
MEAN % OF CD3+CD56+ NKT CELLS
2.50
2.00
1.50
1.00
0.50
0.00
UNTREATED
ETOH
LPS
OLE1
OLE5
OLE10
HT1
HT5
HT10

G

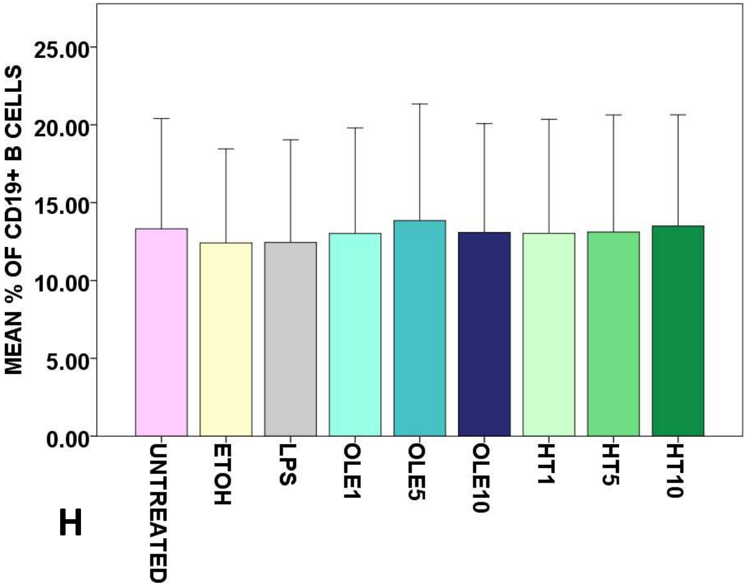
MEAN % OF CD19+ B CELLS
25.00
20.00
15.00
10.00
5.00
0.00
UNTREATED
ETOH
LPS
OLE1
OLE5
OLE10
HT1
HT5
HT10

H

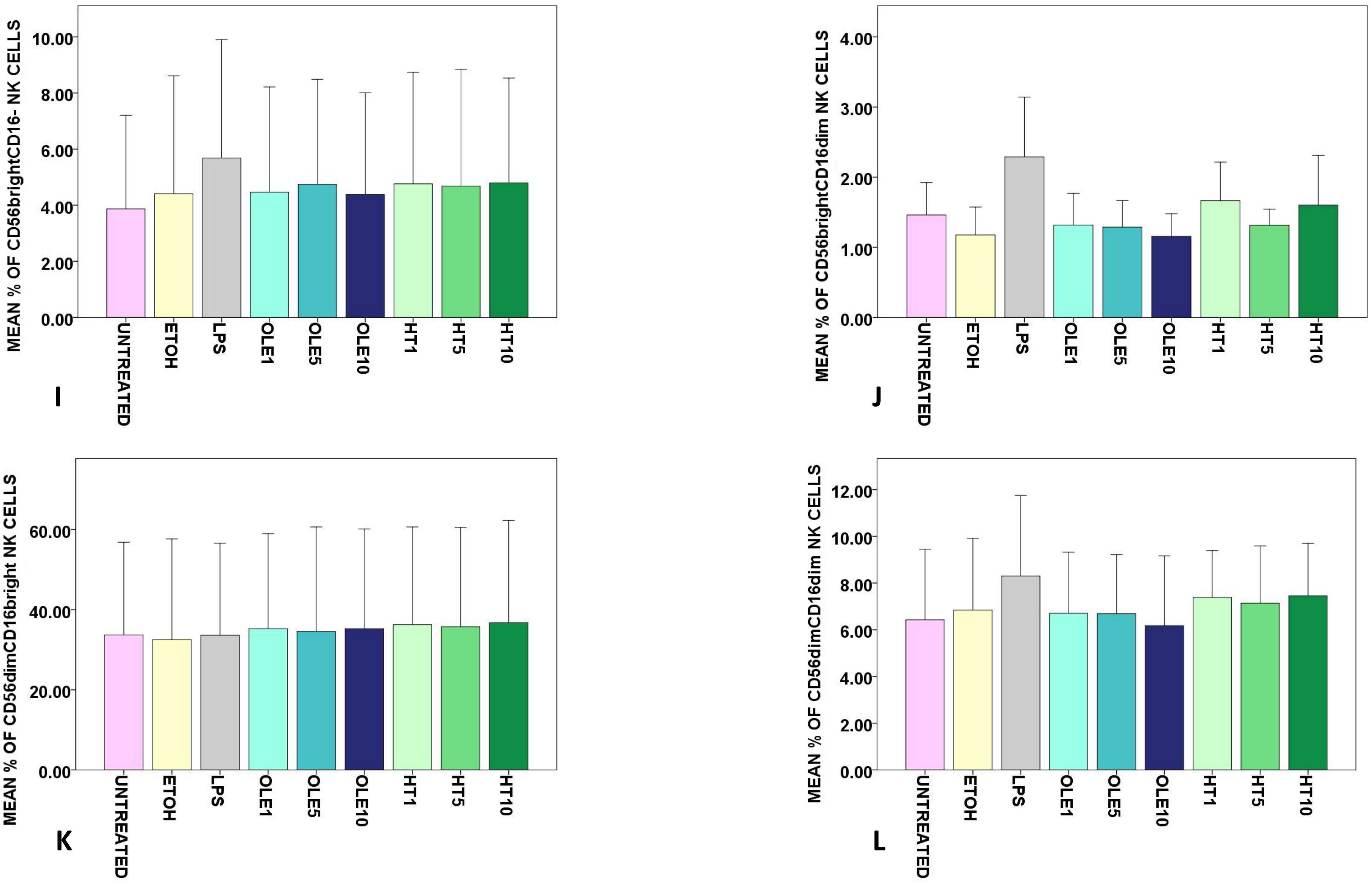

MEAN % OF CD56brightCD16- NK CELLS
10.00
8.00
6.00
4.00
2.00
0.00
UNTREATED
ETOH
LPS
OLE1
OLE5
OLE10
HT1
HT5
HT10
I
MEAN % OF CD56brightCD16dim NK CELLS
4.00
3.00
2.00
1.00
0.00
UNTREATED
ETOH
LPS
OLE1
OLE5
OLE10
HT1
HT5
HT10
J
MEAN % OF CD56dimCD16bright NK CELLS
60.00
40.00
20.00
0.00
UNTREATED
ETOH
LPS
OLE1
OLE5
OLE10
HT1
HT5
HT10
K
MEAN % OF CD56dimCD16dim NK CELLS
12.00
10.00
8.00
6.00
4.00
2.00
0.00
UNTREATED
ETOH
LPS
OLE1
OLE5
OLE10
HT1
HT5
HT10
L

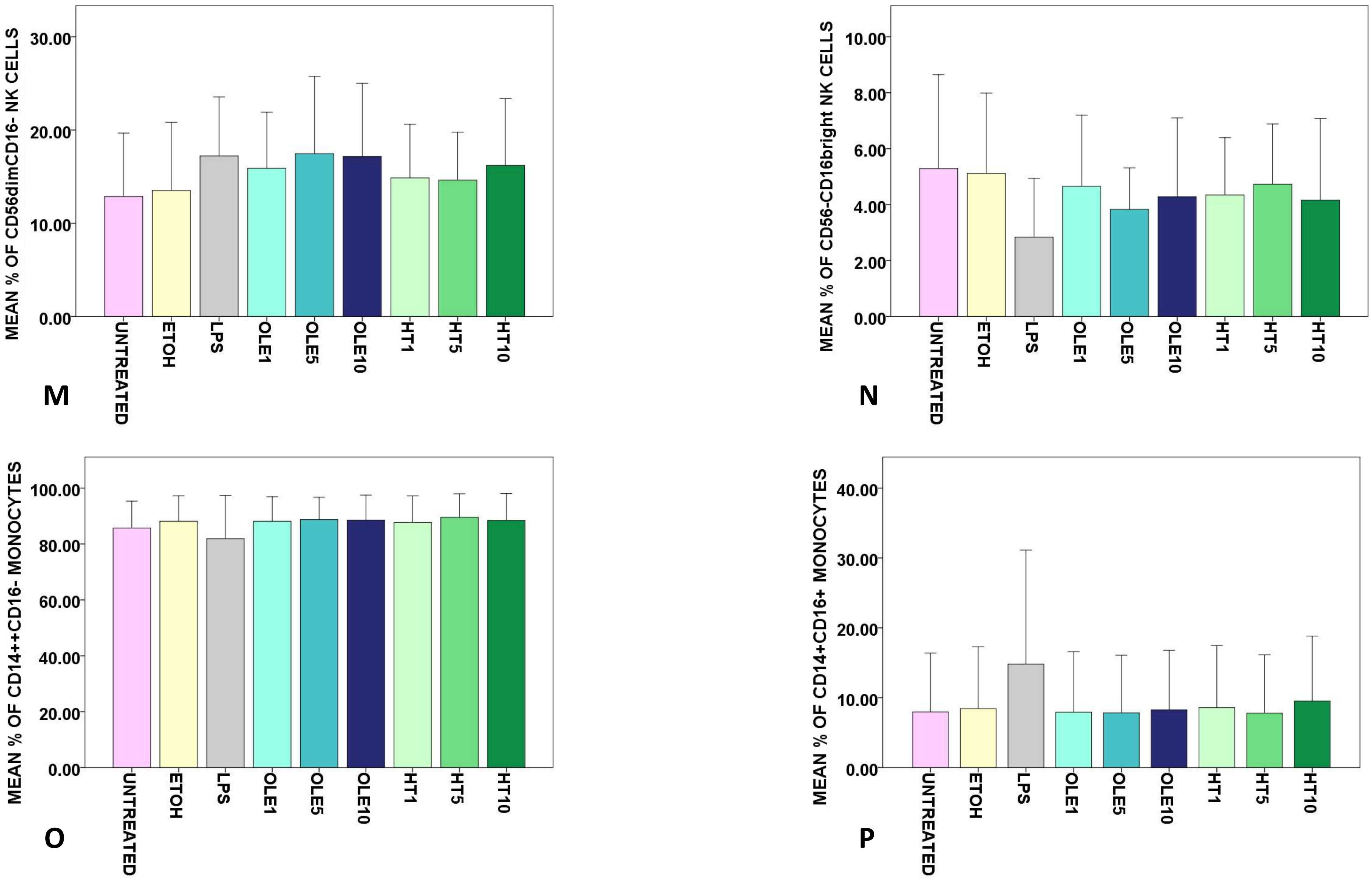
M
MEAN % OF CD56dimCD16- NK CELLS
0.00
10.00
20.00
30.00
UNTREATED
ETOH
LPS
OLE1
OLE5
OLE10
HT1
HT5
HT10
N
MEAN % OF CD56-CD16bright NK CELLS
0.00
2.00
4.00
6.00
8.00
10.00
UNTREATED
ETOH
LPS
OLE1
OLE5
OLE10
HT1
HT5
HT10
O
MEAN % OF CD14++CD16- MONOCYTES
0.00
20.00
40.00
60.00
80.00
100.00
UNTREATED
ETOH
LPS
OLE1
OLE5
OLE10
HT1
HT5
HT10
P
MEAN % OF CD14+CD16+ MONOCYTES
0.00
10.00
20.00
30.00
40.00
UNTREATED
ETOH
LPS
OLE1
OLE5
OLE10
HT1
HT5
HT10

FIGURE S2. **Effects of OLE and HT on the frequency of lymphocyte and monocyte subsets as assayed by flow cytometry on PBMCs from donors of Adult (n=5) group.** Each diagram represent a PBMC population/subset for which only non-significant differences were obtained. The mean percentages of the studied lymphocyte or monocyte subsets recorded for each experimental condition are showed as bars, whereas error bars represent one standard deviation. Panels show data as follows: A, lymphocytes; B, T cells; C, CD4+ T cells; D, CD8+ T cells; E, CD4+CD8+ T cells; F, CD4−CD8− T cells; G, CD3+CD56+ NKT cells; H, B cells; I, CD56brightCD16- NK cells; J, CD56brightCD16dim NK cells; K, CD56dimCD16bright NK cells; L, CD56dimCD16− NK cells; M, CD14++CD16− classical monocytes; N, CD14+CD16+ non-classical monocytes. %, percentage; UNTREATED, cells that received no treatment; ETOH, cells treated only with vehicle (ethanol); LPS, cells treated with 1 µg/mL lypopolysaccharide; OLE1, cells treated with 1 µM oleuropein; OLE5, cells treated with 5 µM oleuropein; OLE10, cells treated with 10 µM oleuropein; HT1, cells treated with 1 µM hydroxytyrosol; HT5, cells treated with 5 µM hydroxytyrosol; HT10, cells treated with 10 µM hydroxytyrosol. SD, standard deviation.

FIGURE S3

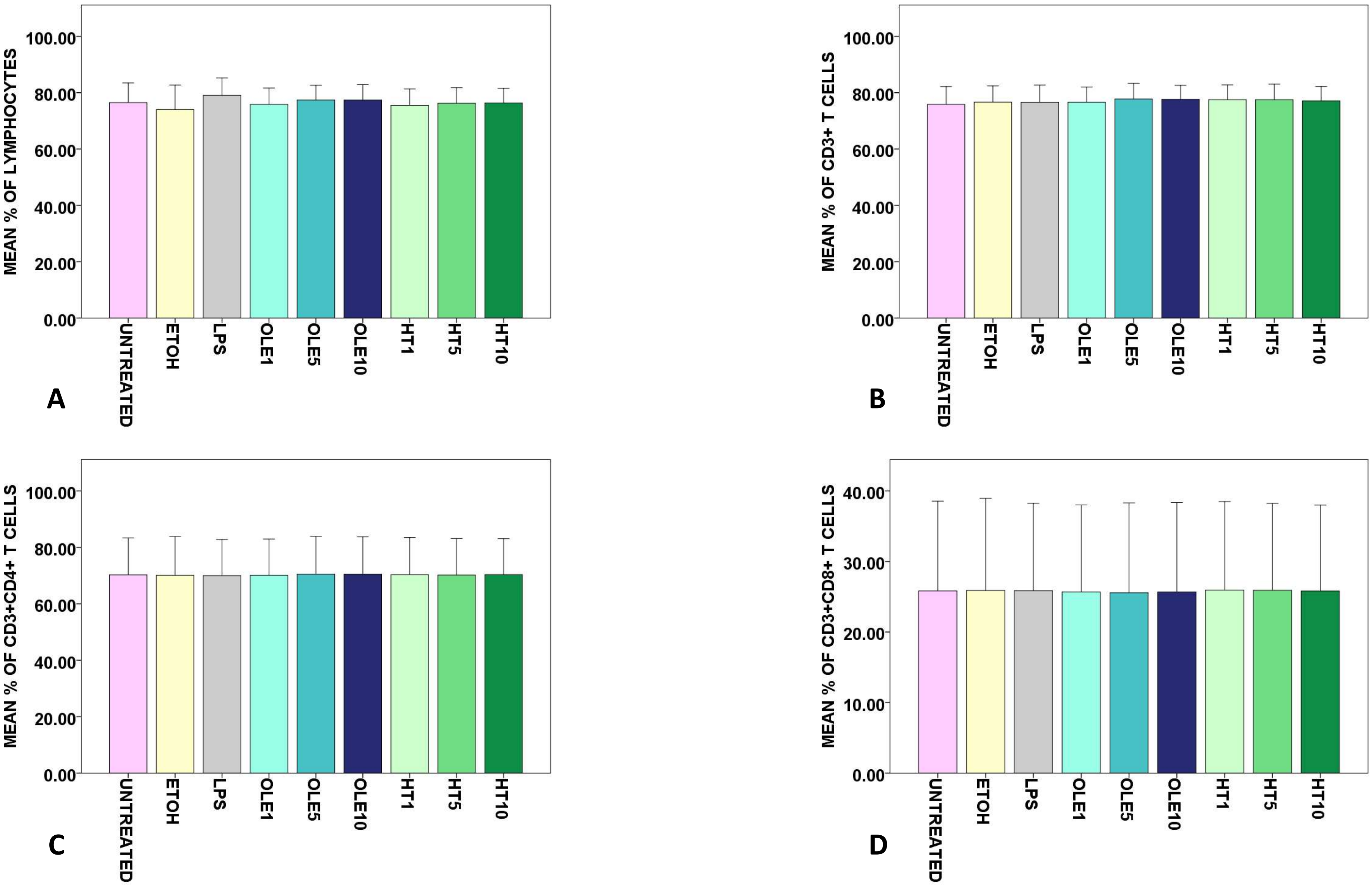
MEAN % OF LYMPHOCYTES
MEAN % OF CD3+ T CELLS
MEAN % OF CD3+CD4+ T CELLS
MEAN % OF CD3+CD8+ T CELLS
0.00
20.00
40.00
60.00
80.00
100.00
10.00
30.00
40.00
UNTREATED
ETOH
LPS
OLE1
OLE5
OLE10
HT1
HT5
HT10
A
B
C
D

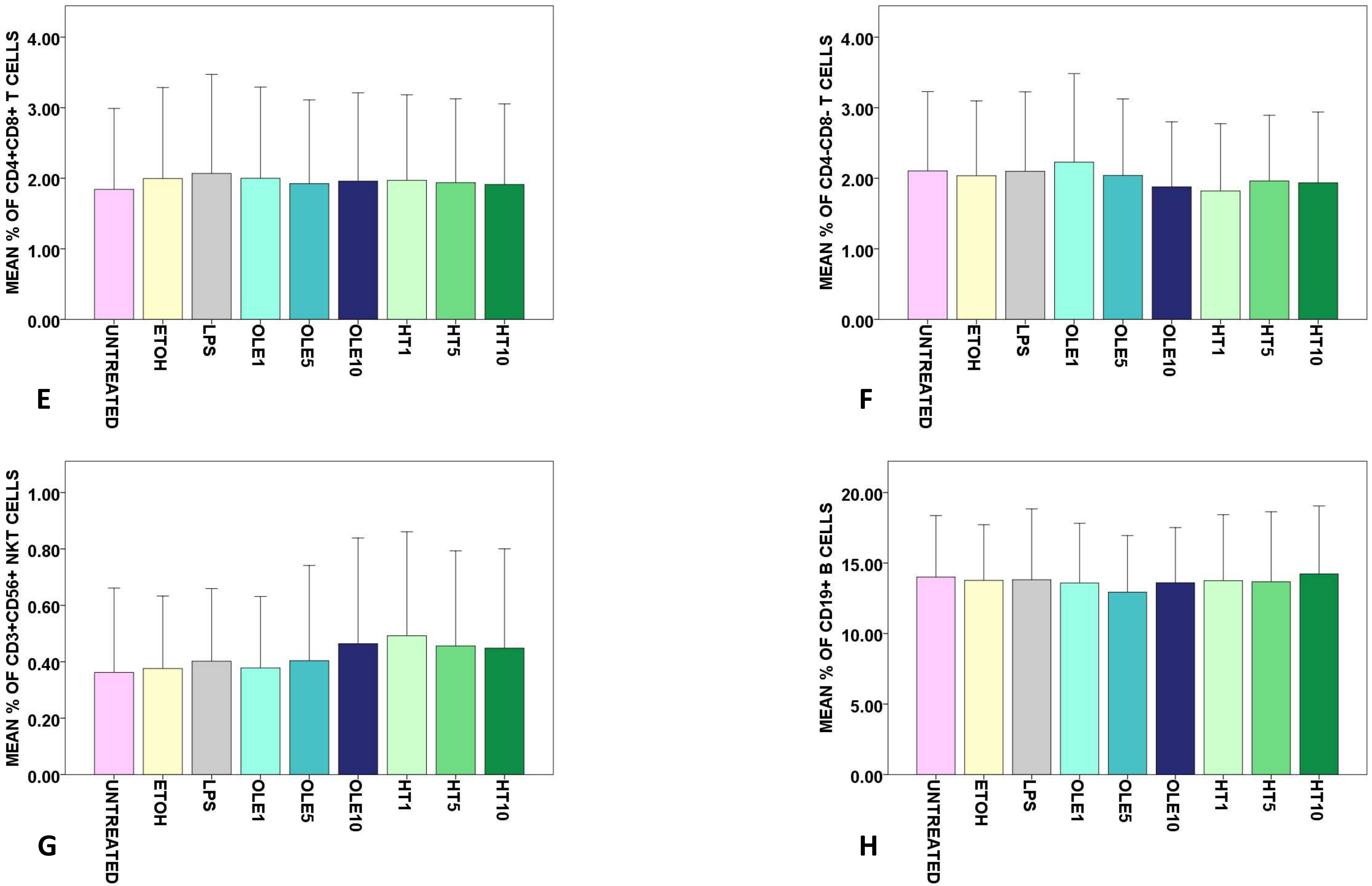
MEAN % OF CD4+CD8+ T CELLS
0.00
1.00
2.00
3.00
4.00
UNTREATED
ETOH
LPS
OLE1
OLE5
OLE10
HT1
HT5
HT10
E
MEAN % OF CD4-CD8- T CELLS
0.00
1.00
2.00
3.00
4.00
UNTREATED
ETOH
LPS
OLE1
OLE5
OLE10
HT1
HT5
HT10
F
MEAN % OF CD3+CD56+ NKT CELLS
0.00
0.20
0.40
0.60
0.80
1.00
UNTREATED
ETOH
LPS
OLE1
OLE5
OLE10
HT1
HT5
HT10
G
MEAN % OF CD19+ B CELLS
0.00
5.00
10.00
15.00
20.00
UNTREATED
ETOH
LPS
OLE1
OLE5
OLE10
HT1
HT5
HT10
H

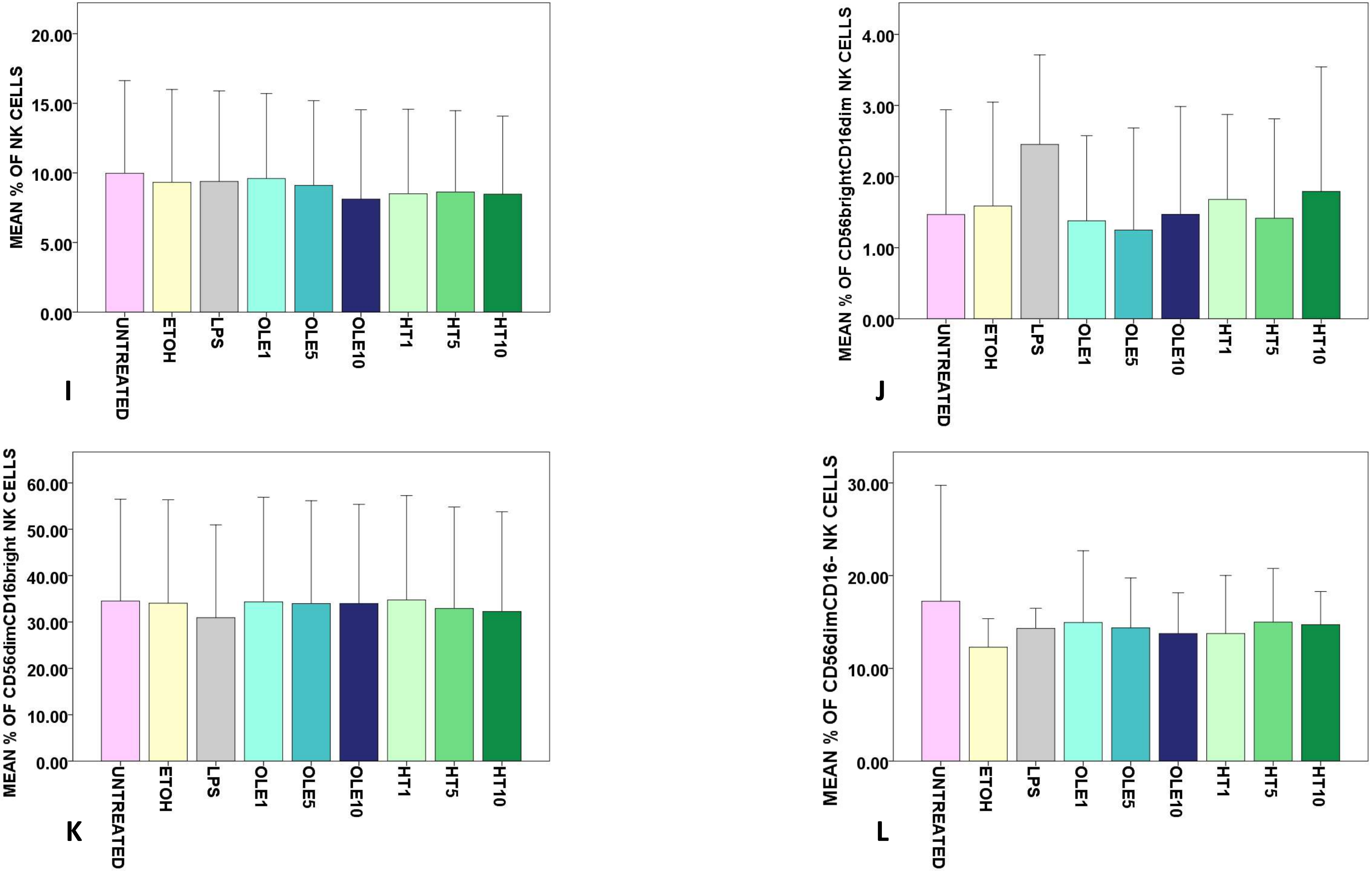
MEAN % OF NK CELLS
20.00
15.00
10.00
5.00
0.00
UNTREATED
ETOH
LPS
OLE1
OLE5
OLE10
HT1
HT5
HT10
I
MEAN % OF CD56brightCD16dim NK CELLS
4.00
3.00
2.00
1.00
0.00
UNTREATED
ETOH
LPS
OLE1
OLE5
OLE10
HT1
HT5
HT10
J
MEAN % OF CD56dimCD16bright NK CELLS
60.00
50.00
40.00
30.00
20.00
10.00
0.00
UNTREATED
ETOH
LPS
OLE1
OLE5
OLE10
HT1
HT5
HT10
K
MEAN % OF CD56dimCD16- NK CELLS
30.00
20.00
10.00
0.00
UNTREATED
ETOH
LPS
OLE1
OLE5
OLE10
HT1
HT5
HT10
L

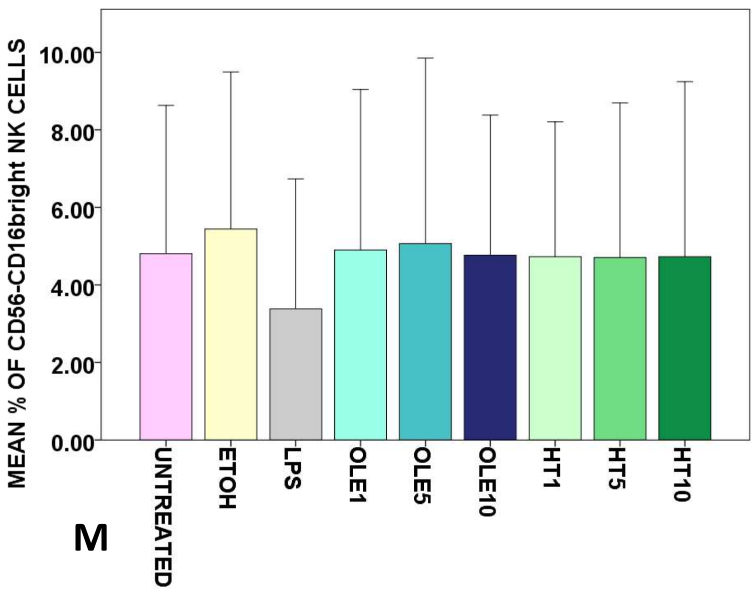
MEAN % OF CD56-CD16bright NK CELLS
10.00
8.00
6.00
4.00
2.00
0.00
UNTREATED
ETOH
LPS
OLE1
OLE5
OLE10
HT1
HT5
HT10

M

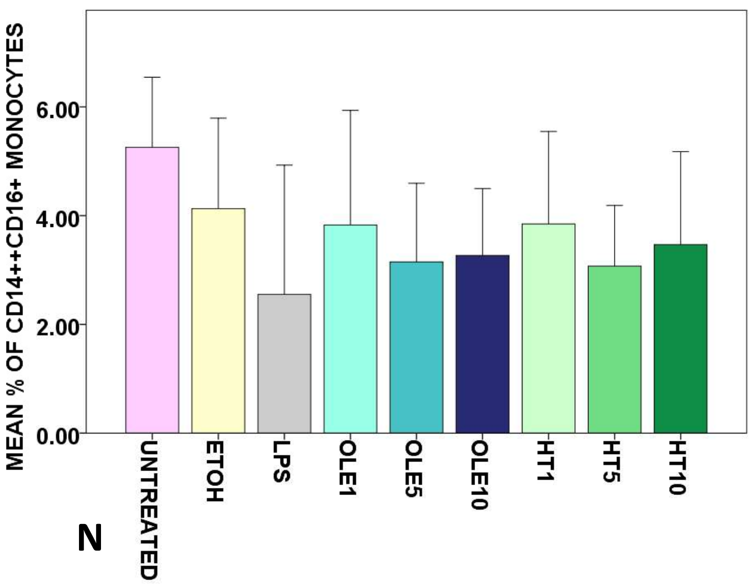
MEAN % OF CD14++CD16+ MONOCYTES
6.00
4.00
2.00
0.00
UNTREATED
ETOH
LPS
OLE1
OLE5
OLE10
HT1
HT5
HT10

N

FIGURE S3. **Effects of OLE and HT on the frequency of lymphocyte and monocyte subsets as assayed by flow cytometry on PBMCs from donors of Senior (n=5) group.** Each diagram represent a PBMC population/subset for which only non-significant differences were obtained. The mean percentages of the studied lymphocyte or monocyte subsets recorded for each experimental condition are showed as bars, whereas error bars represent one standard deviation. Panels show data as follows: A, lymphocytes; B, T cells; C, CD4+ T cells; D, CD8+ T cells; E, CD4+CD8+ T cells; F, CD4−CD8− T cells; G, CD3+CD56+ NKT cells; H, B cells; I, NK cells; J, CD56brightCD16dim NK cells; K, CD56dimCD16bright NK cells; L, CD56dimCD16− NK cells; M, CD56−CD16bright NK cells; N, CD14++CD16+ intermediate monocytes. %, percentage; UNTREATED, cells that received no treatment; ETOH, cells treated only with vehicle (ethanol); LPS, cells treated with 1 µg/mL lypopolysaccharide; OLE1, cells treated with 1 µM oleuropein; OLE5, cells treated with 5 µM oleuropein; OLE10, cells treated with 10 µM oleuropein; HT1, cells treated with 1 µM hydroxytyrosol; HT5, cells treated with 5 µM hydroxytyrosol; HT10, cells treated with 10 µM hydroxytyrosol. SD, standard deviation.
